# Identification of disease ameliorated metabolite candidates from Gut Microbes and their interacting targets based on a novel estimating function

**DOI:** 10.3389/fmicb.2026.1770840

**Published:** 2026-03-10

**Authors:** Hongtu Cui, Zhen Liao, Xia Kuang, Ji-Yun Zhang, Chong-Yuan Song, Yan-Jin Liu, Zhenshun Cheng, Zi-Xin Deng, Feng-Biao Guo

**Affiliations:** 1 Department of Respiratory and Critical Care Medicine, Zhongnan Hospital of Wuhan University, School of Pharmaceutical Sciences, Wuhan University, Wuhan, China; 2 Key Laboratory of Combinatorial Biosynthesis and Drug Discovery, Ministry of Education, Wuhan University, Wuhan, China; 3 Department of Respiratory and Critical Care Medicine, Zhongnan Hospital of Wuhan University, Wuhan, China; 4 Wuhan Research Center for Infectious Diseases and Cancer, Chinese Academy of Medical Sciences, Wuhan, China; 5 Hubei Engineering Center for Infectious Disease Prevention, Control and Treatment, Wuhan, China

**Keywords:** association network, disease-ameliorating metabolites, disease-metabolite association, host disease, metabolites from gut microbiota

## Abstract

The gut microbiota plays a crucial role in human health and the progression of diseases. As key mediators of the interactions between gut microbiota and host diseases, metabolite changes have a significant influence on disease development and progression. However, without experimental validation by metabolomics, identifying the level of metabolite changes in specific diseases remains challenging. For this reason, we devised a scoring function to quantitatively estimate metabolite changes across disease phenotypes by integrating changes in the relative abundance of gut microbes owning these metabolites, and hereby developed the online repository to reserve these associations of metabolites and diseases. The consequent GMMAD with v2.0 currently contains 83 diseases and 6966 metabolites. Among them, 85,136 associations are of statistical significance (paired-sample *t*-test, *p-value* < 0.05 and FDR < 0.1). Notably, the scoring function showed 84.06% consistency with experimentally validated disease-metabolite associations, outperforming the prior semi-qualitative method (72.5%). Aided by this resource, we identified 130 beneficial metabolites across 27 diseases. Among them, literature supports that 48 could ameliorate the disease status in animals or clinical trials. We listed these 48 promising drug molecules on the website. Furthermore, we constructed a metabolite-gene association network and provided information on the origins of metabolites. All the metabolite-associated information would help researchers reveal the mechanisms of how gut microbes regulate disease progression and guide drug development. The GMMAD v2.0 is freely accessible at http://gepa.org.cn/GMMAD2/.

## Graphical Abstract

**Figure:**
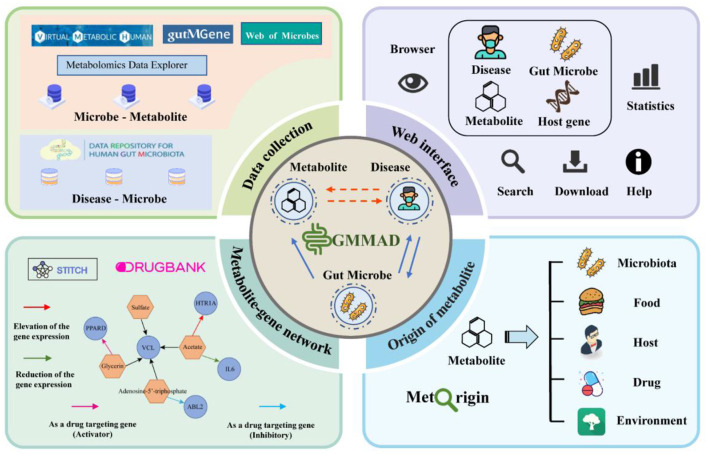

**Highlights**

- Quantifying disease-metabolite changes using a novel microbial abundance-integrated scoring system.
- Integrating disease-gut microbe-metabolite-gene associations with an updated GMMAD v2 database.
- Facilitating drug development via a metabolite-gene association network.
- Providing 130 disease-beneficial metabolites (48 literature-validated) for 27 diseases.

## Introduction

The relationship between the gut microbiota and human health and disease has attracted increasing attention in recent years (Ghosh et al., 2022; Iliev et al., 2025; Kang et al., 2024; Ross et al., 2024; Wang Y. et al., 2023; Wu et al., 2024). Gut microbiota influences various physiological and pathological processes primarily through their metabolites, which are considered key mediators of host health (Krautkramer et al., 2021; Takeuchi et al., 2024). For example, short-chain fatty acids (SCFAs), produced by various gut microbes such as members of the *phyla Bacteroidetes and Firmicutes*, have been shown to regulate host energy metabolism by activating G-protein-coupled receptors (e.g., GPR41 and GPR43), thereby contributing to reduced risks of diabetes and obesity (Cong et al., 2022; Lee D. H. et al., 2024; Mann et al., 2024; Shimizu et al., 2025). In addition, intestinal microbial metabolites trimethylamine N-oxide (TMAO) have been implicated in promoting atherosclerosis, thrombosis, and cardiovascular inflammation by modulating cholesterol metabolism, inhibiting bile acid synthesis, and activating inflammatory signaling pathways, among other mechanisms (Chen et al., 2023; Dolkar et al., 2024; Pathak et al., 2020; Saaoud et al., 2023; Witkowski et al., 2020). The gut microbiota is also closely associated with systemic cancers. For instance, gut-derived amino acids such as methionine have been implicated in tumor initiation and progression by modulating cell growth and tumor suppressor pathways, including S-adenosylmethionine (SAM) metabolism and *TRAIL-R2* expression (Lin K. et al., 2025; Strekalova et al., 2015; Wanders et al., 2020; Xia et al., 2024).

Although numerous studies have elucidated the complex relationship between the gut micro biota and human diseases, most have focused on isolated associations, such as between diseases and microbes or between microbes and metabolites. There remains a lack of a comprehensive and systematic resource that integrates multidimensional associative data encompassing diseases, gut microbes, and their metabolites. To address this gap, we defined a scoring function and accordingly developed the first version of the GMMAD (Gut Microbial Metabolite Association with Disease) data base (Wang C. Y. et al., 2023). While GMMAD 1.0 made significant strides in integrating gut microbial-disease associations, its scoring function employs the qualitative change of gut microbes primarily. In recent years, advancements in sequencing technologies, along with improvements in computational hardware and software, have led to significant increases in the speed and quality of microbial sequencing. These technological developments have facilitated the generation of vast meta genomic and 16 S rRNA sequencing data, providing a robust foundation for studying the associations between diseases and microbes. Furthermore, Wakita et al. observed that the compositional differences between the metabolome and the micro biome were similar, and that the principal component scores of the two groups were significantly correlated, as demonstrated through Principal Component Analysis (PCA) (Wakita et al., 2018). This finding offers a novel perspective for further exploration of the interactions between the micro biome and the metabolome.

In response, we developed the second edition of GMMAD. Compared to the first version, the second edition has seen significant improvements and expansions in the following areas: (a) A more precise association strength scoring system based on the relative abundance data of gut microbiota has been developed to assess the relationships between diseases and metabolites quantitatively. (b) Association data between metabolites and host genes from various databases were integrated, and a metabolite-gene network was constructed, offering a novel perspective for studying the impact of microbial metabolites on host gene expression. (c) Detailed source information for metabolites was recorded to help users better understand the mechanisms of metabolite generation. These enhancements significantly increase the value of the GMMAD database for screening disease biomarkers and drug candidates, as well as understanding the regulating mechanism of gut microbes on specific diseases. The latest version of the database is currently available at http://gepa.org.cn/GMMAD2/. Aided by such a resource, we try to identify ameliorated metabolites in various diseases and confirm them by literature reviews.

## Materials and methods

### Data collection

We systematically integrated resources from multiple databases to comprehensively collect and organize data related to gut micro biota, metabolites, genes, and their interactions. Firstly, the disease names in GMMAD are standardized and organized according to the disease classification system of Medical Subject Headings (MeSH), ensuring consistency and standardization in disease terminology. Microbial annotation data were sourced from the NCBI classification database, which provides a comprehensive taxonomic hierarchy from phylum to species, enabling precise classification and identification of microbes. Metabolite data were obtained from the PubChem database, including detailed ID numbers, KEGG identifiers, SMILES sequences, and molecular formulas. Additionally, metabolite source information from the MetOrigin (Yu et al., 2022) database was integrated, clarifying that metabolites originated from various categories, including host, microbes, food, and drugs. For gene information, we provided gene IDs from the Ensembl, NCBI, HGNC, and UniProt databases, ensuring the comprehensiveness and traceability of the gene data, while facilitating researchers' ability to compare and validate data across different databases.

For disease-microbe information, we integrated data from the GMrepo (Dai et al., 2022) database, which contains 58,903 human intestinal samples (17,618 metagenomic data and 41,285 amplicon data) from 253 experimental projects, covering 92 disease phenotypes. This database provides not only the categories and relative abundance levels (mean, median, variance) of bacterial genera and strains across different disease phenotypes, but also basic information such as sequencing projects, sample sizes, and sample demographics, including age and gender. Ultimately, we compiled data on the relative abundance of strains at various disease stages for 83 diseases and 6,966 microbes.

For microbe-metabolite association information, we retrieved data on metabolites produced by gut microbes from multiple public databases. The Virtual Metabolite Database for Humans (VMH) (Noronha et al., 2019) integrates metabolite, gene, protein, and disease data to support metabolic research and disease mechanism exploration, from which we compiled information on 818 microbes and 2,152 metabolites. The gutMGene (Cheng et al., 2022) database contains experimentally validated relationships between gut microbes, microbial metabolites, and target genes, from which we obtained 1,331 associations involving 332 gut microbes, 207 microbial metabolites, and 222 genes. The WOM (Kosina et al., 2018) database collects three-dimensional data from microbes, metabolites, and the environment, from which we extracted information on 55 microbial strains and 608 metabolites. The Metabolomics Data Explorer data base (Han et al., 2021), developed by Han et al., provides an integrated mass spectrometry process for intestinal microbiota, from which we gathered data on 178 common microbial strains and 357 metabolites produced by them. We organized the data from these databases, standardized metabolite names using the PubChem database to ensure consistency, and removed redundancy, ultimately compiling the association information for 1,322 microbes and 2,944 metabolites.

For metabolite-gene association information, we incorporated data on the regulatory associations between metabolite molecules and genes from the gutMGene (Cheng et al., 2022), STITCH(Kuhn et al., 2010), and Drug Bank (Knox et al., 2024) databases. For the STITCH database, we collected information on the strength of the association between metabolite molecules and genes. For the Drug Bank database, we referenced the interaction types and directions in the DGI database to categorize metabolite-regulated genes as activated, repressed, or indeterminate. Ultimately, we obtained 53,278 metabolite-gene associations involving 903 metabolites and 7,916 genes.

### Disease-metabolite association strength score and confidence score

**Figure 1:**
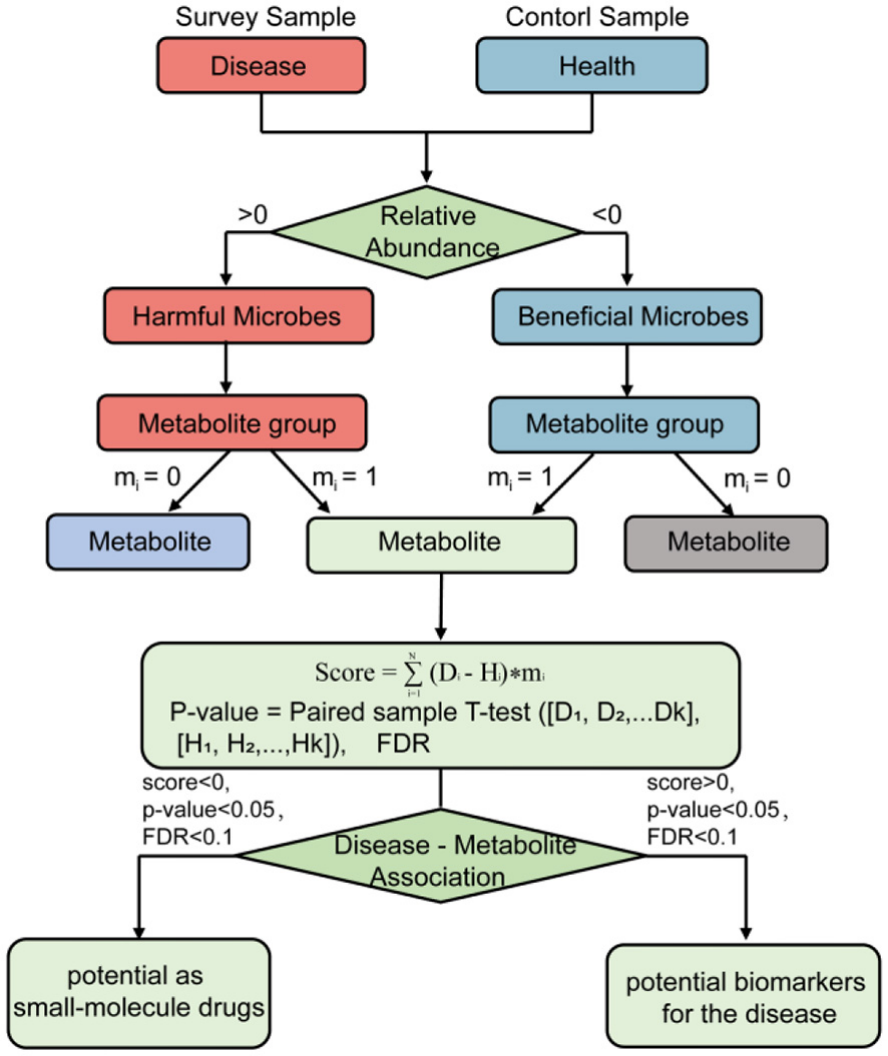
The workflow of disease-metabolite association strength score and confidence score.

Unlike the first version of GMMAD (Wang C. Y. et al., 2023), which was based on qualitative annotations (e.g., labeling microbes as beneficial or harmful in specific diseases), GMMAD v2.0 adopts a quantitative framework that integrates gut micro biome abundance data to evaluate metabolite-level alterations more precisely across disease states. Specifically, we defined two key quantitative metrics: the Association Strength Score and the Confidence Score, to quantify both the strength of the association between diseases and metabolites and the confidence level of these associations (Figure 1).

The strength of the association score is used to quantify changes in the abundance of specific metabolites in a disease state. For each disease metabolite pair, the score is calculated using the following formula:


$$score=\sum_{i=1}^{N}(D_{i\;}-\;H_{i}{)}^{*}\;m_{i}$$

The formula is defined based on our hypothesis and previous observation that the microbial abundance is strongly associated with the levels of almost all its metabolites (Wakita et al., 2018). Therefore, the metabolite changing magnitude should be significantly associated with its host's abundance change. Here, D_i_ and H_i_ represent the strain-level abundance of the i-th microbe in the disease and healthy states, respectively; *N* denotes the total number of microbial species involved (i.e., strains that may produce the target metabolite); and *m*_*i*_ is a binary indicator (1 if the i-th strain produces the target metabolite, 0 if not). This design ensures only metabolically relevant strains (those producing the target metabolite) contribute to the score, avoiding interference from irrelevant microbes and enabling the score to accurately reflect microbe-driven metabolite changes.

It is important to clarify that the association strength score was defined per disease metabolite pair, not per sample pair. For each disease condition and each metabolite, we first aggregated the micro biome data across all samples within the disease group and all samples within the healthy control group. Specifically, for each microbial species, we calculated its median relative abundance across all samples in the disease group and across all samples in the healthy control group. These median values were then used as D_i_ and H_i_ in the formula to compute a single association strength score for each metabolite disease pair. The median was chosen because it provides a robust estimate of central tendency that is less sensitive to extreme values and individual-level fluctuations, which is particularly important given the compositional and zero-inflated nature of micro biome data. Since most of the microbial-metabolite data collected in the previous work are at the strain level, and the abundance data of the micro biota provided by different diseases are at the species level, this study assumes that the abundance data for D_i_ and H_i_ in this equation are numerically equivalent to the species-level abundance data.

To assess the reliability of the scores, this study further introduced a paired-sample *t-test* to evaluate the confidence of the association strength scores, which was calculated as:


$$\begin{array}{l} \text{P-value}=paired\;sample\;T\;test\left(\left[{\text{D}}_{1},{\text{D}}_{2},...{\text{D}}_{\text{k}}\right],\left[{\text{H}}_{1},{\text{H}}_{2},...\;{\text{H}}_{\text{k}}\right]\right) \end{array}$$

Here, k represents the total number of species included in the calculation (matching the species-level data used to proxy D_i_ and H_i_), and D_1_…D_k_, H_1_…H_k_ denotes the median species-level abundances of these k species in the disease and healthy states, respectively. Prior to applying the *t-test*, we verified the normality of the paired differences using the Shapiro-Wilk test in R, which confirmed a normal distribution (*p-value* > 0.05). To control for false positive results arising from multiple statistical tests, we applied the Benjamini-Hochberg procedure to calculate the FDR for each test result.

In this study, we evaluated changes in metabolite levels and their potential medicinal or biomarker value in disease patients based on the metabolite score (score), the confidence level (*p-value*) and FDR. When the metabolite score is less than 0, it indicates that the metabolite is metabolized at a lower level in disease patients compared to the healthy population; conversely, when the score is greater than 0, it suggests that the metabolite is metabolized at a higher level. Moreover, for metabolites with a score less than 0, a *p-value* less than 0.05, and an FDR less than 0.1, we consider them to have potential as small-molecule drugs for treating the disease. For metabolites with a high absolute score ranking, *p-value* less than 0.05, and FDR less than 0.1, we consider them to be potential biomarkers for the disease.

### Identification of disease-associated beneficial metabolites based on negative correlations

Based on the above criteria, we screened beneficial metabolites across various disease states. Within each disease group, metabolites were ranked based on their scores, and those with a score < 0 and a significant *p-value* were selected. The top five metabolites with the most negative scores were considered beneficial metabolites. These metabolites exhibited significantly reduced metabolism in patients, suggesting potential therapeutic benefits.

### Database construction

The GMMAD v2.0 database is built using the Django 1.11 framework, which offers excellent scalability and a robust architecture. The front-end is developed using HTML, CSS, and Bootstrap to ensure a responsive layout, while the back-end is built on Python 3.9. Data is stored using MySQL 5.6.50. Additionally, GMMAD v2.0 integrates the Cytoscape plugin for visualizing metabolite-gene association networks and supports high-definition downloads of visualization images. The database has been tested for compatibility with Internet Explorer and Chrome browsers on Windows operating systems to ensure optimal display. It is currently accessible via http://gepa.org.cn/GMMAD2/.

### Data analysis and visualization

All statistical analyses and visualizations were performed in R (version 4.3.1) using the package data. table, dplyr, tidyr, and ggplot2. Additionally, the network was drawn using Cytoscape (version 3.10.2).

## Results

### Scoring method comparison, predictive accuracy validation of two GMMAD database versions, and novel disease-metabolite association predictions in GMMAD v2

**Figure 2:**
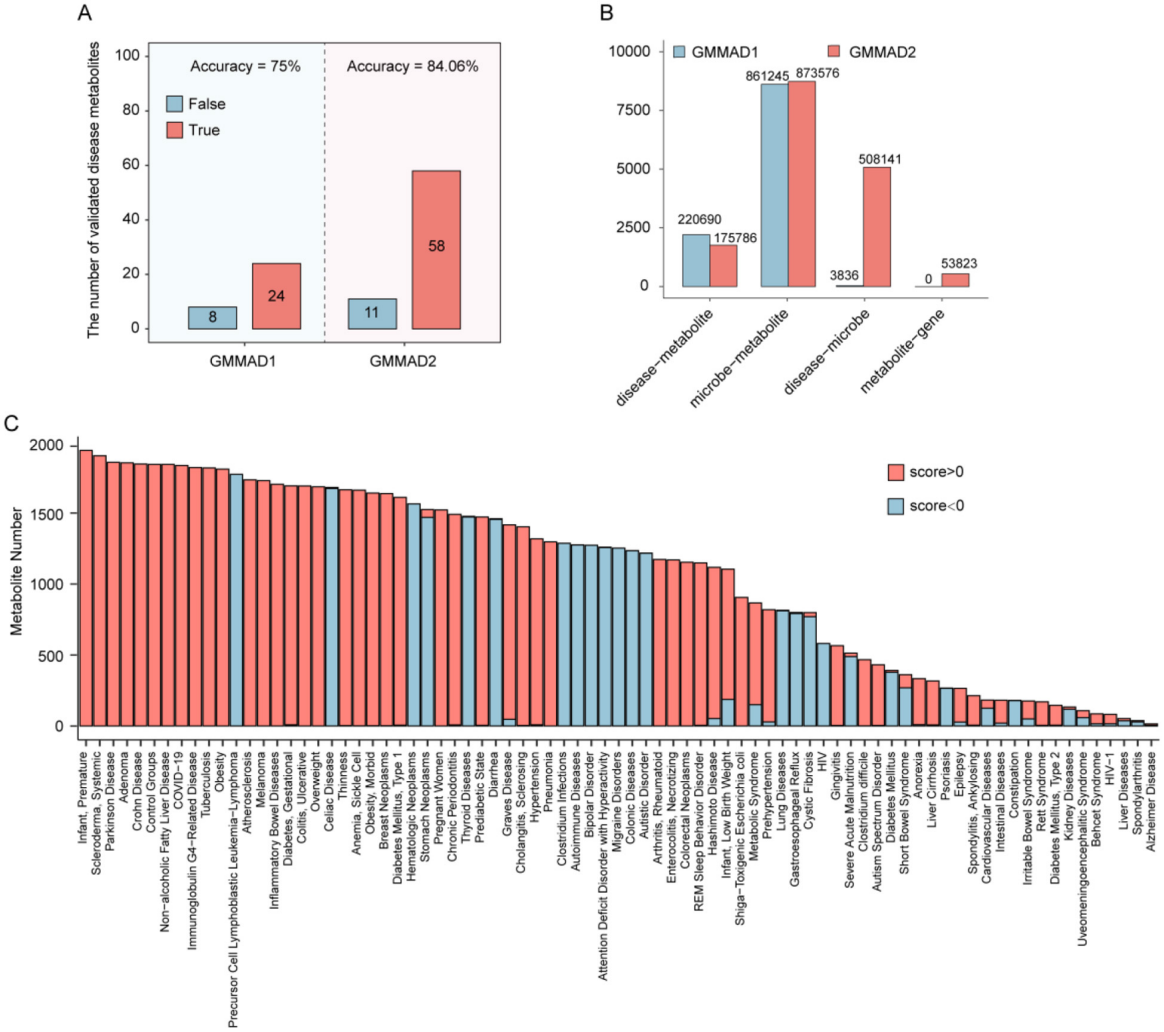
**(A)** Comparison of the predictive accuracy between the two versions of the scoring method. “True” indicates predictions consistent with literature-reported associations, while “False” indicates predictions in the opposite direction. **(B)** Comparison of the number of disease–metabolite, disease-microbe, microbe–metabolite, and metabolite–gene associations in both versions of the database. **(C)** Score Distribution of significant associations across 79 different diseases.

Compared with GMMAD v1.0, which only calculated disease-metabolite association scores qualitatively, GMMAD v2 implements two critical improvements to overcome the limitations of qualitative approaches (e.g., inability to quantify the “strength of associations”). First, we upgraded the scoring logic to a quantitative framework: using microbial relative abundance as the core input to compute numerical scores, enabling precise characterization of differences in association strength between distinct diseases and metabolites. Previously, we regarded all microbial species associated with specific diseases as having equal abundance and uniquely differentiated them in their altering (association) direction (Wang C. Y. et al., 2023). Therefore, metabolite-altering strength is only determined by the number of species increasing vs decreasing. Second, we expanded the dataset size of disease-microbe and microbe-metabolite interactions, providing more robust support for the reliability of the scores (Figure 2B).

To validate the reliability and predictive efficacy of the quantitative microbial abundance-based scoring method, this study systematically evaluated it through multidimensional optimization and comparison with experimental data. Given the high variance inherent in microbial abundance data, we chose to use the median rather than the mean to calculate metabolite level scores, minimizing the influence of outliers. The algorithm was further optimized by introducing a sample size threshold and ranking the degree of abundance change. When the sample size was ≥ 5, and the top 100 microbes based on abundance change were selected, the score was 84.06%, consistent with the direction of disease-metabolite associations that had been experimentally validated, which was significantly better than the 72.5% of the previous semi-qualitative scoring method. (Supplementary Table S1, Figure 2A).

Building on this, we predicted 175,786 disease-metabolite association entries covering 79 diseases and 2,436 metabolites (Figure 2B). Applying a strict statistical threshold (paired-sample *t-test, p-value* < 0.05 and false discovery rate (FDR) < 0.1), we identified 85,136 significantly associated entries, encompassing 79 diseases and 2,148 metabolites (Figure 2C).

### Database Content

**Figure 3:**
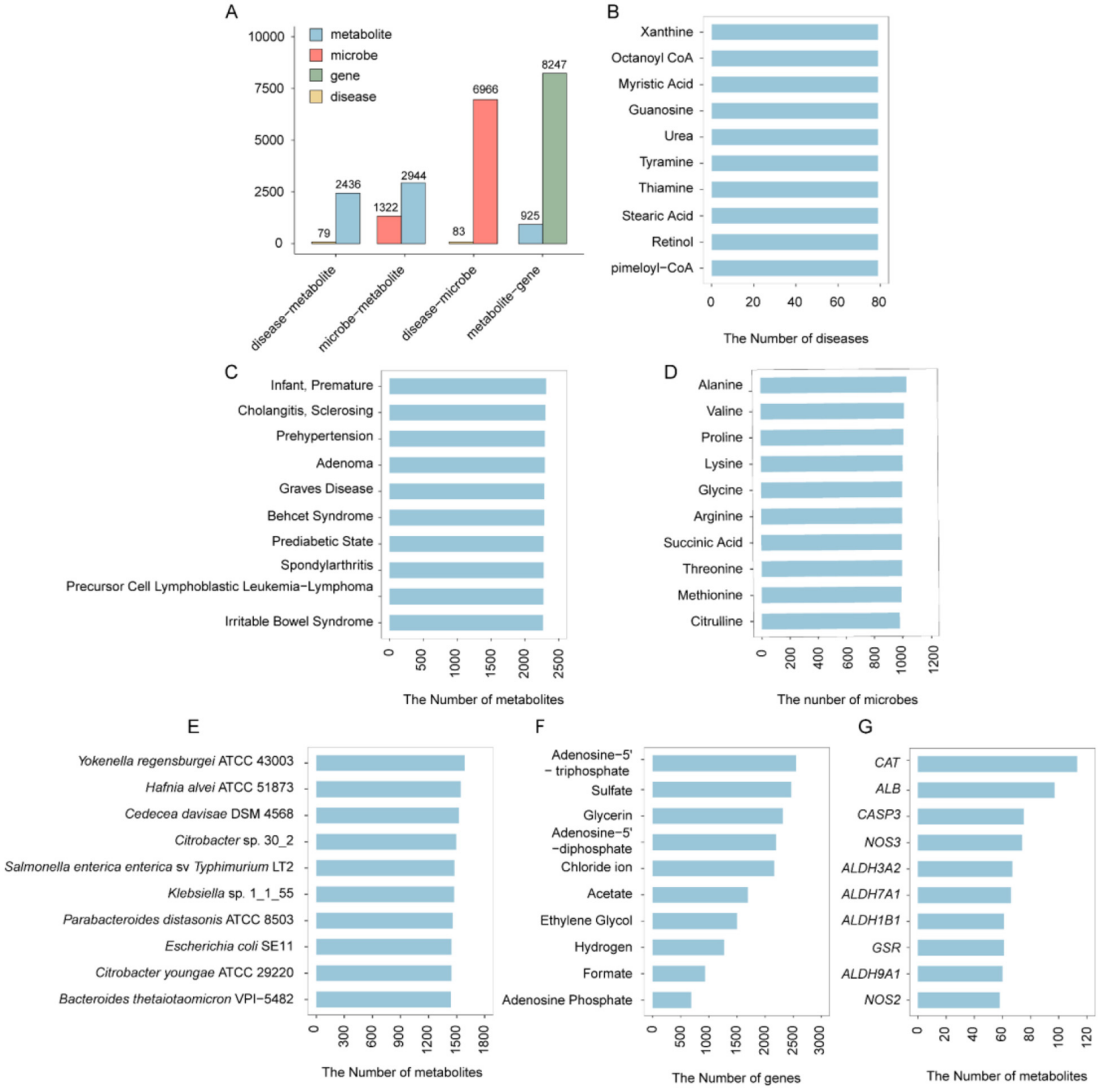
Overview of the GMMAD Database. **(A)** Total counts of unique metabolites, microbes, genes, and diseases included in the database. **(B)** Top 10 metabolites ranked by the number of associated diseases in disease–metabolite associations. In fact, these 10 metabolites have significant associations with the same number of diseases. **(C)** Top 10 diseases ranked by the number of associated metabolites in disease–metabolite associations. **(D)** Top 10 metabolites ranked by the number of associated microbes in microbe–metabolite associations. **(E)** Top 10 microbes ranked by the number of associated metabolites in microbe–metabolite associations. **(F)** Top 10 metabolites ranked by the number of associated genes in metabolite–gene associations. **(G)** Top 10 genes ranked by the number of associated metabolites in metabolite–gene associations.

The GMMAD v2.0 database has undergone comprehensive updates and expansions compared to the first version, resulting in a significant increase in data volume and enhanced functionality. (1) GMMAD v2.0 currently includes 175,786 disease-metabolite associations (spanning 79 diseases and 2,436 metabolites), 873,576 microbe-metabolite associations (involving 1,322 microbes and 2,944 metabolites), and 508,141 disease-microbe associations (covering 83 diseases and 6,966 microbes). (2) A new Metabolite-Gene Associations module has been introduced, encompassing 53,638 metabolite-gene associations (covering 925 metabolites and 8,232 genes) (Figures 2C, 3A). (3) GMMAD v2.0 incorporates information on the strains involved in disease-metabolite associations, offering users more detailed data on metabolite-disease relationships. (4) The database now includes information on the source of each metabolite, allowing users to determine whether the metabolite is endogenously produced by the host or exogenously acquired. (5) It provides basic structural information for each metabolite, such as SMILES sequences and molecular two-dimensional structures. (6) The Metabolite-Gene Function module evaluates the binding affinity between metabolites and genes and integrates the Cytoscape visualization plugin to graphically depict the targeting relationships between metabolites and genes. (7) The database also provides standardized IDs for diseases, microbes, metabolites, and genes, which can be linked to authoritative databases such as MeSH, Taxonomy, PubChem, and Ensembl, allowing users to access more detailed information.

We analyzed the top 10 metabolites, diseases, genes, microbes, and in different types of associations (Figures 3B-G). In disease-metabolite associations, xanthine, guanosine, and urea were the metabolites associated with the highest number of diseases, and premature infant was the disease linked to the greatest number of metabolites, suggesting that the disease may involve complex metabolic disturbances (Figures 3B, C).In microbe-metabolite associations, alanine was the metabolite linked to the greatest number of microbes, while *Yokenella regensburgei* ATCC 43003 was the microbe associated with the most metabolites (Figures 3D, E). In metabolite-gene interactions, adenosine5′-triphosphate was the metabolite associated with the most genes, and *CAT* was the gene most frequently linked to metabolites, suggesting that this gene may play a key role in metabolic regulation (Figures 3F, G).

### GMMAD user interface

GMMAD v2.0 provides an intuitive interface for efficiently browsing, searching, and downloading association data between diseases, microbes, metabolites, and genes. The database organizes the data into four main association categories: disease-metabolite, disease-microbe, microbe-metabolite, and metabolite-gene. Each category is further subdivided into relevant subcategories, such as “Disease,” “Gut Microbes,” “Metabolites,” and “Genes.”

**Figure 4:**
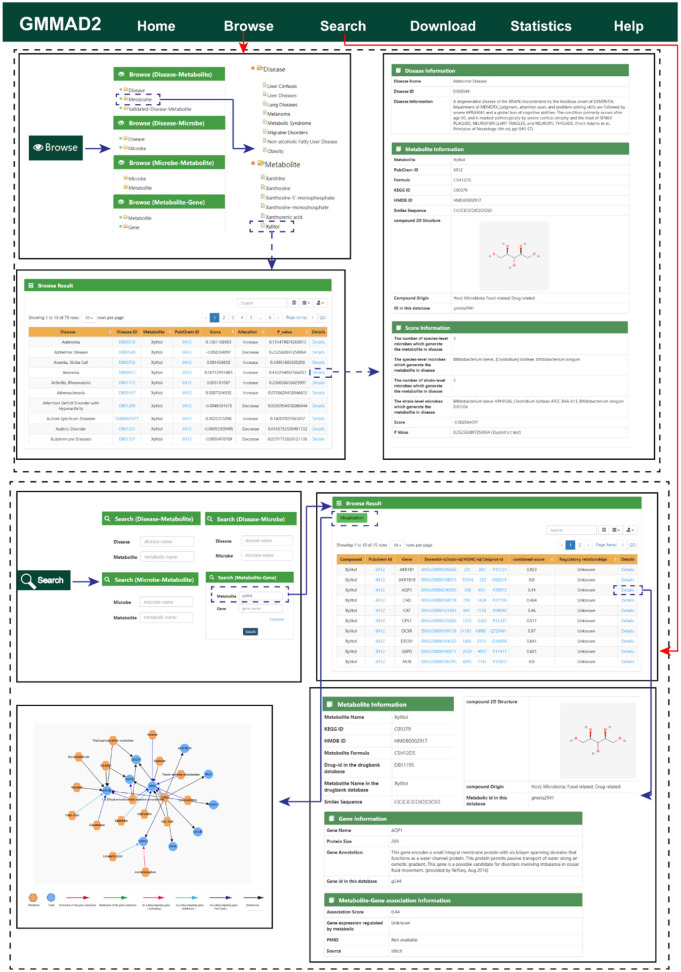
Schematic diagram of GMMAD's browse and search interfaces. The top panel is about the browse function, whereas the bottom is for the search function.

In the browsing interface, users can easily visualize the hierarchical structure of different categories and their subcategories using the tree browser. For instance, users can expand the “Diseases - Metabolites” category to explore further its subcategories, such as “Diseases” and “Metabolites”. Upon selecting a term within a subcategory, the related associations are displayed in a table on the right-hand side, as illustrated in Figure 4 for disease-metabolite associations. For example, selecting the metabolite xylitol will display its scores across different diseases in a table, each row representing a specific association and a brief description. The identifiers for metabolites and diseases are linked to external databases (e.g., PubChem, NCBI), allowing users to click on these links for detailed information about the relevant entities. In the results table, clicking on the “details” link provides additional information about the row, such as the source of the metabolite, its molecular formula and structural data, and details on the microbes involved in calculating the score.

In the search interface, users can query association relationships by entering the name or ID of diseases, microbes, metabolites, and genes. For example, in the case of metabolite-gene associations, selecting the xylitol metabolite and clicking “search” will display the strength and direction of the association between the metabolite and its target gene in a tabular format. Each row represents a specific association, along with a brief description. The identifiers for metabolites and genes are linked to external databases (e.g., PubChem, Ensembl, and UniProt), allowing users to click on these links for detailed information about the relevant entities. In the results table, clicking the “Details” link provides additional information for the selected row, such as the metabolite sources and detailed gene information. For metabolite-target gene associations, clicking the “Visualize” button generates a metabolite-gene network diagram illustrating the target relationships, offering users a more intuitive understanding of the associations. Additionally, users can download the search results by clicking the icon located at the top right corner of the page. All tutorials and detailed instructions are provided on the “Help” page, ensuring users can quickly get started and fully utilize the program.

### Identification of beneficial metabolites across different diseases

We systematically analyzed all diseases in the GMMAD v2.0 database to pick out the top five beneficial metabolites of negative associations (Supplementary Table S2), excluding those without statistical significance (*p-value* > 0.05 or FDR > 0.1). Among the total 79 diseases, 27 have literature-reported metabolite-intervening trials, and they have 130 statistically significant and beneficial metabolite associations. After the literature review, 50 metabolites got information on whether they are observably beneficial effects in animal or clinical intervention trials. Only two reported cases contradict our identification, and this suggests our identification has the precision of 48/50 = 96%. Table 1 presents some representative disease cases supported by the literature and shows consistent predictions. Based on the site of disease occurrence, these diseases can be categorized into digestive, liver, respiratory, hematological, cardiovascular, immune system, endocrine, oral, neurological, and other diseases. Detailed literature review results for all 48 metabolites with available intervention information, including full references and evidence summaries, are available in the Supplementary Materials Section.

**Table 1:** Beneficial metabolites supported by literatures across different diseases.

**Disease**	**Metabolic**	**Score**	**P-Value**	**FDR**	**PMID**
Alzheimer disease	3-Hydroxybutyric acid	−0.02668	0.0434	0.08228	33803253
Anemia, sickle cell	Butyrate	−0.0851	0.04993	0.0924	26719803
Anorexia	Butyrate	−0.06997	0.00564	0.01562	36675302
Autistic disorder	Cysteine	−0.20618	0.00117	0.00431	34658941
Autoimmune diseases	Phenylalanine	−0.1158	0.00004	0.00027	34523675
Autoimmune diseases	Nicotinic acid	−0.11209	0.00001	0.00005	37845835
Bipolar disorder	Leucine	−0.14976	0.00012	0.00065	27179557
Bipolar disorder	Phenylalanine	−0.14853	0.00006	0.00037	3944066
Bipolar disorder	l-Isoleucine	−0.14124	0.00012	0.00067	27179557
Cardiovascular diseases	Pantothenic acid	−0.09165	0.02316	0.04985	38949913
Cardiovascular diseases	Tryptophan	−0.08984	0.02381	0.05079	28179491
Cardiovascular diseases	Nicotinamide	−0.08817	0.02988	0.06073	33568522
Colonic diseases	Succinic acid	−0.21583	0.0002	0.00102	24856584
Colonic diseases	Arginine	−0.21234	0.00029	0.00141	doi: 10.1016/j.jff.2018.11.019
Colonic diseases	Lysine	−0.20924	0.00038	0.00177	38351003
Colonic diseases	Alanine	−0.20911	0.0004	0.00181	37898769
Colonic diseases	Valine	−0.20756	0.0004	0.00183	9468343
Cystic fibrosis	Stearic acid	−0.15414	0.0032	0.00982	33025706
Cystic fibrosis	Aspartic acid	−0.15413	0.00346	0.01047	32974221
Diabetes mellitus	Indole	−0.05788	0.00007	0.00043	34192642
Diabetes mellitus, type 2	Taurocholic acid	−0.04509	0.01971	0.04402	30298865
Diabetes, gestational	Daidzein	−0.005	0.01754	0.04	32506178
Diabetes, gestational	Genistein	−0.00238	0.00088	0.00346	32506178
Diabetes, gestational	Glycitein	−0.00238	0.00088	0.00346	32506178
Diarrhea	Alanine	−0.1475	0.00006	0.00035	2007961
Diarrhea	Tryptophan	−0.14226	0.00003	0.00023	38877403
Diarrhea	Nicotinic acid	−0.14183	0.00003	0.0002	207728
Gastroesophageal reflux	Arginine	−0.14756	0.00178	0.00606	22207112
Gastroesophageal reflux	Glycine	−0.1459	0.00116	0.0043	22207112
Gingivitis	D-Arabinose	−0.00281	0.04324	0.08207	35866308
Graves disease	Melatonin	−0.06502	2.68E-07	4.3e-06	27374868
HIV	Spermidine	−0.27103	3.73E-08	8.06e-07	37406129
Hematologic neoplasms	Arginine	−0.28095	9.18E-10	4.04e-08	27745970
Hypertension	Lactulose	−0.02006	0.04882	0.09066	30825362
Irritable bowel syndrome	Chenodeoxycholic acid	−0.02308	0.04046	0.07768	28666948
Liver cirrhosis	Daidzein	−0.01555	1.87E-06	0.00002	37780202
Liver cirrhosis	Cholecalciferol	−0.00699	0.02147	0.04684	30271082
Liver diseases	Taurocholic acid	−0.03012	0.03481	0.06891	35199809
Liver diseases	Dextrin	−0.02768	0.00013	0.00072	21936050
Lung diseases	Nicotinic acid	−0.0866	0.00231	0.00748	22375599
Lung diseases	Inosine	−0.0844	0.00403	0.0118	36313960
Metabolic syndrome	Spermine	−0.08449	0.00017	0.00088	24530553
Metabolic syndrome	Linolenic Acid	−0.0819	0.00003	0.00021	22894911
Migraine disorders	Nicotinic acid	−0.10174	0.00002	0.00017	12934790
Stomach neoplasms	Oleic Acid	−0.28251	1.21E-08	3.15e-07	24823908
Stomach neoplasms	Alanine	−0.27636	7.68E-08	1.5e-06	25622826
Stomach neoplasms	Arginine	−0.27426	9.25E-08	1.76e-06	19036482
Thyroid diseases	Leucine	−0.17368	0.00001	9.87e-05	30524299

**Figure 5:**
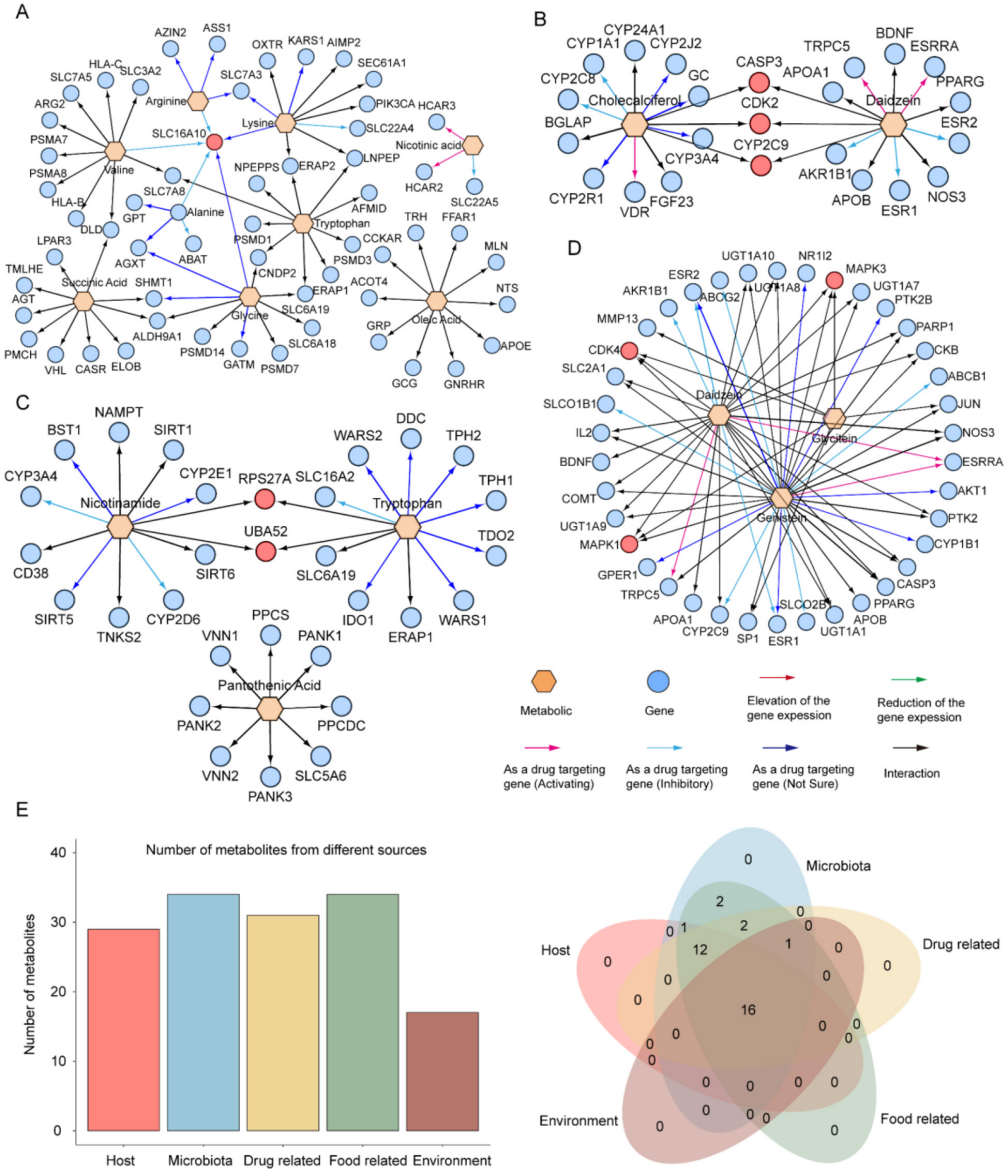
Metabolite-gene association network in **(A)** digestive diseases, **(B)** liver Cirrhosis, **(C)** cardiovascular disease, and **(D)** gestational diabetes mellitus. **(E)** Origin analysis of identified metabolites.

To further explore the molecular mechanisms, we constructed metabolite-gene association networks linked to the diseases mentioned above. For digestive diseases, we found that the *SLC16A10* gene is the core gene of the network (Figure 5A). As a gene encoding an aromatic amino acid transporter, *SLC16A10* may influence digestive diseases by regulating the intestinal absorption and metabolism of amino acids (Halestrap, 2013; Mariotta et al., 2012), making it a key target for exploring metabolic mechanisms in digestive diseases. For liver diseases, daidzein and cholecalciferol simultaneously targeted *CYP2C9, CASP3*, and *CDK2* (Figure 5B). Previous studies have shown that *CASP3* deficiency reduces pro-fibrotic gene expression (Thapaliya et al., 2014), while *CDK2* deficiency decreases liver fibrosis and downregulates *ACTA2* and *COLLA1* (Otto et al., 2023), suggesting that targeting these genes may offer therapeutic strategies for liver fibrosis. For cardiovascular diseases, pantothenic acid, tryptophan, and nicotinamide co-targeted *RPS27A* and *UBA5* (Figure 5C). Notably, *RPS27A* is significantly upregulated in patients with idiopathic pulmonary hypertension, indicating its involvement in disease progression (Huang et al., 2023). For gestational diabetes mellitus, daidzein, genistein, and glycitein target *MAPK1, MAPK3*, and *CDK4* (Figure 5D). These genes play crucial roles in GDM pathogenesis, with MAPK signaling affecting placental angiogenesis and *CDK4* down regulation impairing pancreatic β-cell proliferation (Nazari et al., 2017; Subiabre et al., 2017). For bipolar disorder, leucine, isoleucine, and phenylalanine collectively target *PSMD14* (Supplementary Figure 1), a gene recently implicated as a potential therapeutic target for this condition (O'Connell et al., 2025).

Finally, we conducted a comprehensive traceability analysis of all the aforementioned metabolites by integrating the metabolite origin annotation function of the MetOrigin (Yu et al., 2022) database. The results revealed that these metabolites originate from five major sources: endogenous synthesis by the host (human), microbial metabolism, dietary intake, metabolic derivatives from pharmacological interventions, and exogenous substances introduced through environmental exposure (Figure 5E). Notably, there is substantial co-metabolism among metabolites from different sources, and their interactions may collectively influence the host's metabolic state. Therefore, when designing metabolite-based intervention strategies for disease treatment, it is essential to consider the diverse origins of metabolites and their potential synergistic effects to achieve more precise and effective metabolic regulation.

### Disease-metabolite association network identifies alanine as a potential biomarker

**Figure 6:**
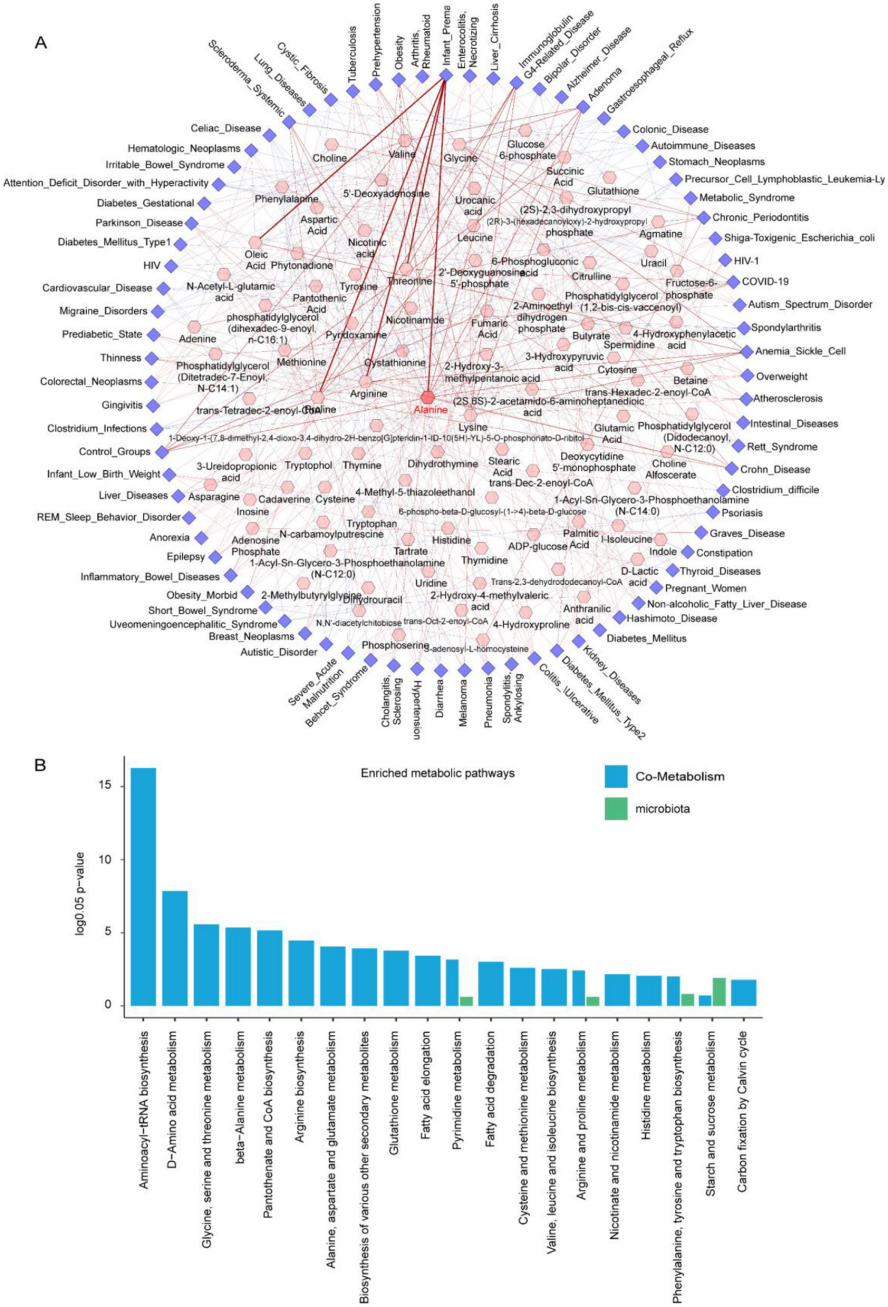
**(A)** Disease–metabolite association network across 79 diseases, highlighting the top five significantly altered metabolites in each disease. The width of the edge represents the strength of the association, and the color of the edge indicates the direction of the association: red for positive association and blue for negative ones. **(B)** The top 20 Enriched metabolic pathways. Metabolic pathways with log 0.05 *P* values greater than 1 are considered statistically significant.

Based on changes in metabolite abundance across different disease states, we selected the top five metabolites showing the most significant alterations for each disease and constructed a disease–metabolite association network (Figure 6A). In this network, the thickness of each edge represents the strength of the association between a metabolite and a disease. By integrating both the connectivity and association strength of each node, we calculated a weighted average score to assess the potential involvement of each metabolite across multiple diseases (Supplementary Table S3). Our results showed that Alanine had the highest connectivity, suggesting its broad involvement in various disease processes. In contrast, Threonine exhibited lower connectivity but demonstrated strong association strengths within its linked diseases, ranking first in the weighted score. This indicates that Threonine may play critical roles in specific disease contexts. Notably, Alanine also ranked highly in the weighted scores, further supporting its potential role in disease onset and progression. Previous studies have identified Alanine as a biomarker for several diseases, including diabetes, sepsis, prostate cancer, glioma, and COVID-19 (Alcicek et al., 2024; Kimura-Ohba et al., 2023; Lee K. S. et al., 2024; Liu et al., 2024; Tessem et al., 2008), which aligns well with our findings and highlights its promise in clinical diagnosis and prognosis assessment.

Based on these results, we further performed metabolite enrichment analysis, which revealed that the identified metabolites were significantly enriched in amino acid metabolism pathways (Figure 6B). This finding highlights the central role of amino acid metabolism in gut micro biota dysbiosis and provides a theoretical foundation for elucidating the mechanisms underlying metabolic disorders associated with micro biota-related diseases, as well as for developing targeted therapeutic strategies.

## Discussion

In recent years, an increasing number of studies have highlighted the significant role of gut metabolites in regulating human health (Arifuzzaman et al., 2024; Gao et al., 2025; Jiang et al., 2025; Song et al., 2025). For instance, indole-3-propionic acid (IPA) alleviates intestinal mucosal inflammation in inflammatory bowel disease (IBD) by binding to Heat Shock Protein 70 (HSP70) and inducing apoptosis of Th1/Th17 cells, thereby modulating mucosal immune responses (Gao et al., 2025). Hippuric acid plays a key role in regulating host metabolic health and modulating diabetes progression by mediating gut microbial interactions and lowering circulating uric acid to improve glucose tolerance (Wu et al., 2025). However, current research resources predominantly focus on disease-microbe associations, with a lack of comprehensive databases addressing the relationship between diseases and metabolites (Wang et al., 2022). Monitoring metabolite changes in diseases is a highly complex and time-consuming process, often relying on metabolomics technologies for accurate analysis. The GMMAD (Wang C. Y. et al., 2023) database was developed to accelerate the identification and analysis of disease-associated metabolites by integrating public databases and literature-extracted datasets of disease-microbe and microbe-metabolite interactions. The database leverages data on the relative abundance of microbes in diseased and healthy populations to predict trends in metabolite changes, thereby providing insights into the role of various metabolites in disease onset and progression. The tool is designed to assist researchers in identifying potentially beneficial small molecules, offering new perspectives and targets for drug discovery, and accelerating the drug development process.

The primary function of the GMMAD v2.0 database is to predict metabolite level changes in disease states. Data on the relative abundance of microbes at the species level across diseases are extracted from GMrepo (Dai et al., 2022). On the other hand, GMMAD v2.0 integrates four microbial-metabolite databases [VMH(Noronha et al.,2019), gutMGene (Cheng et al., 2022), WOM (Kosina et al., 2018), and Metabolomics Data Explorer (Han et al., 2021)], covering 1,322 microbes and 2,944 metabolites. By quantifying changes in microbial abundance, GMMAD v2.0 predicts changing trends for 2,436 metabolites across 79 diseases. The association strength score for each metabolite–disease pair is computed by integrating the direction and magnitude of abundance changes of all microbial taxa capable of producing or consuming that metabolite. However, a core assumption underlying this scoring system is that of a linear correlation between microbial abundance and metabolite levels—i.e., that greater abundance of a given taxon necessarily translates into proportionally higher production or metabolic activity. In reality, this linearity may not hold, as microbial metabolic output is influenced by multiple factors, including gene expression levels, substrate availability, interspecies interactions, and environmental conditions (Schreiber and Ackermann, 2020). Moreover, the model currently assumes equal contribution from all bacterial taxa associated with a metabolite, without weighting for differences in metabolic efficiency, pathway flux, or ecological roles. These simplifications, while necessary for large-scale integration, may lead to inaccuracies in prediction and should be considered when interpreting the results.

Indeed, in some disease–metabolite associations, the predicted results were not entirely consistent with previously reported findings. For instance, multiple studies have demonstrated that Proline levels are significantly elevated in gastric cancer (Chen et al., 2010; Guszczyn and Sobolewski, 2004), whereas our model predicted a decrease. Upon further analysis, we found that among the microbes involved in the prediction, *Helicobacter pylori* and *Lactobacillus* were significantly increased in gastric cancer patients (Li et al., 2021; Wong et al., 2021), which aligns with existing literature. In contrast, *Anaplasma* spp. were predicted to be decreased in gastric cancer, which contradicts reported findings (Dai et al., 2021), thus contributing to the overall deviation of the prediction from the actual biological observations (Supplementary Figure 2). This discrepancy highlights how inconsistencies in microbial abundance data, combined with the equal-contribution assumption, can propagate into metabolite-level predictions. Moreover, it is important to emphasize that the disease–metabolite scores generated by our model represent correlation rather than causation. From a causal perspective, it remains unclear whether disease onset leads to changes in metabolite levels or if alterations in metabolite levels contribute to disease development. This remains a common challenge in micro biome research. Anyway, these top metabolites with both negative and positive associations deserve to be verified experimentally. If disease status is verified to be the reason for the negative associations, at least these metabolites, as well as those with positive associations, could still be taken as a biomarker of such disease. These metabolites, luckily verified as regulator could be the preferences of clinical trials.

In current disease prediction studies, considerable efforts have focused on constructing machine learning models for disease classification using micro biome features (Geng et al., 2024; Li et al., 2025; Su et al., 2024). However, the relationship between microbes and diseases is often highly complex. Different microbes may exert their biological effects through shared metabolic pathways or common metabolites (Krautkramer et al., 2021). Therefore, models built solely on microbial features may overlook the critical mediating role of metabolites, thereby limiting both predictive accuracy and biological interpretability. In contrast, metabolites—being direct products of microbial and host metabolic activities—are more likely to reflect disease onset and progression through their abundance changes (Jaye et al., 2022; Krautkramer et al., 2021). As such, disease prediction models based on metabolite profiles hold greater potential for practical application and clinical translation. With the rapid advancement of metagenomics and 16S rRNA sequencing technologies, GMMAD v2.0 will continue to expand the spectrum of covered diseases and further integrate high-quality gut microbial metabolic data. Future iterations will aim to refine the scoring algorithm by incorporating taxon-specific metabolic capacities and, where possible, gene-level functional data, thereby moving beyond the equal-contribution assumption and improving the biological fidelity of predictions.

The GMMAD v2.0 database provides information on metabolite-gene associations. Currently, the metabolite-gene association network is derived from the powerful integration of three databases: STITCH (Kuhn et al., 2010), gutMGene (Cheng et al., 2022), and Drug Bank (Knox et al., 2024), which also include drug-target information. For metabolite data, we provide detailed origin categorization for each metabolite based on the Metorigin (Yu et al., 2022) database. This categorization encompasses multi-dimensional data, including host, microbiota, co-metabolism, food sources, drug sources, and environmental sources, facilitating in-depth exploration of the contributions of different organisms in the metabolic process. However, the current metabolite-gene association network focuses primarily on metabolites with strong associations and available drug-target information, without fully considering the role of microbes within this network. In the future, we plan to further integrate microbial data to construct a comprehensive association network that includes diseases, microbes, metabolites, and genes, thereby enhancing the understanding of the relationship between gut micro biota dysbiosis and human diseases.

It is important to note that the GMMAD v2.0 database is designed to bridge the gap between diseases and metabolites, aiming to identify beneficial small-molecule metabolites based on changes in their levels to treat diseases. However, the level of metabolite changes in disease phenotypes, whether increased or decreased, is dynamic and may vary significantly between different populations and geographic regions (Lin C. et al., 2025; Peng et al., 2024; Reynolds et al., 2023). Experimental approaches are still required to validate these metabolite level changes further before practical utilization. In conclusion, we have effectively utilized microbial abundance data to update GMMAD at a quantitative level. Moving forward, we plan to establish closer interconnections with other related resource databases and refine our analytical methods to provide stronger support for elucidating the regulatory mechanisms of gut micro biota on human health and disease.

## Conclusion

GMMAD v2.0 is a database designed for the quantitative analysis of gut microbial metabolite-disease associations, integrating metabolite-gene networks and metabolite source information. Compared to its predecessor, GMMAD 2.0 introduces significant improvements in the quantitative evaluation of disease-metabolite associations, the construction of metabolite-gene networks, and the documentation of metabolite source information. Currently, GMMAD 2.0 includes 85,136 metabolite-disease associations of statistical significance (Paired-sample *t-test, p-value* < 0.05). Forty-eight metabolites were verified to be regulators of ameliorating human disease after literature reviews, and they are promising drug candidates for these diseases, given that they are natural products of gut organisms and have relatively higher safety. Additionally, a new metabolite-gene association module has been incorporated, encompassing more than 50,000 metabolite-gene associations. These advancements substantially enhance the utility of GMMAD for screening disease markers and drug candidates. In the future, GMMAD 2.0 will continue to expand its data, optimize its algorithms, and develop a comprehensive association network linking diseases, microbes, metabolites, and genes, furthering our understanding of how gut microbiota regulates human diseases.

## Data Availability

The data utilized in this study and GMMAD v2.0 are publicly accessible at http://gepa.org.cn/GMMAD2/.
