# Supplementary material for: Identification of disease ameliorated metabolite candidates from Gut Microbes and their interacting targets based on a novel estimating function
Source: Front Microbiol. 2026 Mar 10;17:1770840. doi: 10.3389/fmicb.2026.1770840 (PMC13014617; doi:10.3389/fmicb.2026.1770840)
Supplement: Supplementary file 1 [file Data_Sheet_1.docx]

**Identification of disease ameliorated metabolite candidates from Gut Microbes and their interacting targets based on a novel estimating function**

**Supplementary Section**

**Identification of Beneficial Metabolites Across Different Diseases**

**Digestive diseases**

Anorexia is a chronic eating disorder. Correlational studies have shown that dietary butyrate inhibits the release of pro-inflammatory factors and enhances positive appetite regulation. In experiments with weaned rabbits, butyrate reduced anorexia and increased mean daily weight gain post-weaning(Zhang et al.,2023).

Colon disease, as a major condition of the digestive system, poses a serious threat to public health. A study by Haraguchi et al. found that a specific dose of succinic acid inhibited the proliferation of colon cancer cells and the migration of endothelial cells, suggesting that succinic acid may have potential preventive and therapeutic effects on colon cancer(Haraguchi et al.,2014). Zhang et al. demonstrated using mouse colitis and cell culture models that lysine, at concentrations similar to those found in the intestines of *Dubosiella newyorkensis*-colonized mice, could regulate the regulatory T cell (Treg)/ T helper 17 cell (Th17) balance, inhibit inflammation, reduce colon cell apoptosis, and potentially prevent and control colitis and mucosal damage(Zhang et al.,2024). Wang et al. found, through Mendelian randomization analysis, that alanine was negatively associated with the risk of colon cancer. They suggested that dietary interventions aimed at increasing alanine intake may be a potential strategy for the prevention and treatment of colon adenocarcinoma(Y. Wang et al.,2023). Andrade et al. constructed a mouse model of colitis and found that, when administered at specific doses, arginine significantly attenuated intestinal bleeding, reduced intestinal permeability, and bacterial translocation, showing potential therapeutic effects on colitis in mice(Andrade et al.,2019). Another study demonstrated that a specific dose of valine slowed the growth of human colon cancer cells (HT-29) by altering polyamine metabolism(Selamnia et al.,1998).

Diarrhea is an intestinal disease caused by multiple pathogens and factors. In a study involving elderly patients with severe secretory diarrhea caused by cholera or enterotoxigenic *Escherichia coli,* oral rehydration salts containing 90 mmol/L alanine and 90 mmol/L glucose demonstrated significantly improved absorption and reduced fecal output by 40% compared to standard World Health Organization formulations(Ribeiro et al.,1991). Li et al. investigated the role and underlying mechanisms of tryptophan in alleviating diarrhea using both cellular and animal models. They found that tryptophan mitigates diarrhea by repairing the intestinal barrier via the angiotensin-converting enzyme 2 (ACE2)/amino acid transporter B⁰AT1 axis, and by regulating the mechanistic target of rapamycin (mTOR) and aryl hydrocarbon receptor (AhR) signaling pathways(Li et al.,2024). Turjman et al., using a rabbit jejunal loop model, demonstrated that nicotinic acid, whether administered subcutaneously or intraluminally, effectively prevented cholera toxin-induced intestinal secretion and cyclic AMP (cAMP) elevation. Moreover, it reversed symptoms that had already manifested, suggesting a preventive and therapeutic potential of nicotinic acid against cholera-associated diarrhea(Turjman et al.,1978).

Gastroesophageal reflux (GER) is a condition characterized by the retrograde movement of gastroduodenal contents into the esophagus, leading to discomfort and/or complications, and affecting approximately 15–20% of the global population(Chapelle et al.,2021). Studies have demonstrated that both arginine and glycine protect the esophageal mucosa and alleviate inflammatory damage in GER disease, and in a rat model, these amino acids were shown to dose-dependently reduce the severity of esophageal injury(Nagahama et al.,2012).

Irritable bowel syndrome (IBS) is a common gastrointestinal disorder characterized by abdominal discomfort and abnormal bowel movements. The main subtypes of IBS include diarrhea-predominant (IBS-D), constipation-predominant (IBS-C), and mixed (IBS-M). In a retrospective cohort study, Vijayvargiya et al. (Vijayvargiya et al.,2018)found that patients with IBS-C had reduced levels and synthesis of fecal bile acids. Further clinical trials have confirmed that short-term interventions with delayed-release chenodeoxycholic acid in patients with IBS-C are effective in accelerating colonic transit and alleviating clinical symptoms. In contrast, another study found a significant increase in fecal chenodeoxycholic acid levels in IBS-D patients(Dior et al.,2016), suggesting that chenodeoxycholic acid has a biphasic role in intestinal diseases. Therefore, understanding the mechanism of action of chenodeoxycholic acid in different types of irritable bowel syndrome is crucial for achieving precise treatment of intestinal diseases. These results suggest that the researchers should classify IBS samples into subgroups when performing sequencing detection of gut microbiota.

Stomach neoplasm ranks as the fifth most common malignancy globally. According to the latest cancer statistics in 2024, over 1 million new cases of gastric cancer were reported worldwide, resulting in more than 760,000 deaths(Bray et al.,2024). Oleic acid has been reported to inhibit the growth and survival of cancer cells in low-metastatic cancer cell lines, including the gastric cancer cell line SGC-7901 and the breast cancer cell line MCF-7(Li et al.,2014). Gu et al. demonstrated that alanine suppressed the proliferation of gastric cancer cell lines SGC-7901, AGS, and MKN45 in vitro. Notably, from 54 hours onward, alanine significantly reduced both the total and stabilized cell populations in SGC-7901 cells, thereby impeding cell growth and altering cell status(Gu et al.,2015). In addition, Nanthakumaran et al. found that arginine inhibited the growth of the gastric cancer cell line AGS, increased its apoptotic index, and exerted anti-tumor effects by upregulating the expression of the apoptosis-related gene *CASP8*(Nanthakumaran et al.,2009).

Interestingly, we constructed a metabolite-gene association network linked to the diseases mentioned above, and found that the *SLC16A10* gene is the core gene of the network (Figure 5A). As a gene encoding an aromatic amino acid transporter, *SLC16A10* may influence digestive diseases by regulating the intestinal absorption and metabolism of amino acids(Halestrap,2013; Mariotta et al.,2012). Therefore, *SLC16A10* could serve as a key target for exploring the metabolic mechanisms underlying digestive diseases.

**Liver diseases**

Liver disease represents a major global public health challenge, contributing to approximately 2 million deaths annually and accounting for around 4% of all global mortality(Gan et al.,2025). Taurocholic Acid (TCA), a conjugated bile acid, exerts hepatoprotective effects primarily through modulation of the gut microbiota. It has been reported that a high-fat diet suppresses the abundance of bile salt hydrolase (BSH)-producing bacteria in the gut, leading to reduced levels of unconjugated bile acids, hepatic lipid accumulation, and impaired liver function. In contrast, TCA supplementation significantly reshapes the composition of the intestinal microbiota, increases the abundance of BSH-positive bacteria, and thereby elevates the levels of unconjugated bile acids. This, in turn, activates the Takeda G - protein - coupled Receptor 5 (TGR5) and Farnesoid X Receptor (FXR) signaling pathways, enhances bile acid transport and enterohepatic circulation, expands the bile acid pool, and ultimately ameliorates hepatic lipid metabolism disorders and restores liver function(Xu et al.,2022). Dextrin, a low molecular weight soluble dietary fiber, has also demonstrated promising hepatoprotective potential in animal studies. Kozmus et al.(Kozmus et al.,2011) reported that dietary supplementation with 10% dextrin in a rat model significantly reduced hepatic protein carbonylation levels, increased the ratio of reduced glutathione (GSH) to oxidized glutathione disulfide (GSSG), and alleviated oxidative stress. Moreover, dextrin markedly lowered serum triglyceride and urea levels, indicating its capacity to improve systemic metabolic status and maintain liver functional homeostasis.

Cirrhosis is a terminal stage of chronic liver disease, characterized by progressive hepatic fibrosis and irreversible structural remodeling of the liver, ultimately resulting in liver failure. Daidzein is a natural isoflavonoid that exhibits notable antioxidant and anti-inflammatory properties. Studies have demonstrated that daidzein effectively attenuates inflammatory responses by activating peroxisome proliferator-activated receptors (PPARs) and inhibiting the Nuclear Factor kappa-light-chain-enhancer of Activated B Cells (NF-κB) signaling pathway, thereby reducing the expression of pro-inflammatory cytokines such as monocyte chemoattractant protein-1 (MCP-1/Ccl2) and interleukin-6 (IL-6). In addition, daidzein lowers the level of malondialdehyde (MDA), a lipid peroxidation marker, and restores the activity of superoxide dismutase (SOD), thereby alleviating oxidative stress(Ubaid et al.,2023). Moreover, cholecalciferol exerts a protective effect in cirrhosis. Cirrhotic patients frequently experience vitamin D deficiency, which may be closely related to reduced 25-hydroxylase activity due to hepatic decompensation(Arteh et al.,2010; Sun et al.,2021). Supplementation with cholecalciferol may help slow disease progression by inhibiting the activation of hepatic stellate cells, mitigating fibrosis, and modulating immune-inflammatory responses, thus improving hepatocyte metabolic status(Huang et al.,2018).

More importantly, based on metabolite-gene network analysis, we found that daidzein and Cholecalciferol simultaneously targeted the *CYP2C9*, *CASP3*, and *CDK2* genes (Figure 5B). Previous studies have demonstrated that *CASP3* (-/-) mice exhibit significantly reduced transcriptional expression of pro-fibrotic genes compared to wild-type mice on a Methionine/Choline Deficient (MCD) diet(Thapaliya et al.,2014). This suggests that small-molecule drugs targeting the inhibition of the *CASP3* gene may effectively reduce hepatocyte apoptosis and fibrosis. Similarly, for the *CDK2* gene, it was found that *CDK2*-deficient mice exhibited significantly reduced liver fibrosis and significantly downregulated expression of *ACTA2* and *COLLA1*, which are indicators of liver fibrosis, compared to wild-type control mice(Otto et al.,2023). This implies that the development of targeted inhibitors against the *CDK2* gene could represent an effective strategy for the treatment of liver fibrosis.

**Respiratory diseases**

Lung disease is a collective term for a group of disorders characterized by structural and functional abnormalities of the lungs. Lin et al.(Lin et al.,2012) reported that nicotinic acid administration significantly alleviated lung injury in a rat model by inhibiting the expression of inducible nitric oxide synthase (iNOS) and the activity of poly(ADP-ribose) polymerase (PARP). These results suggest that nicotinic acid may have therapeutic potential in the treatment of lung injury. Wang et al.(N. Wang et al.,2023) demonstrated in animal studies that inosine can indirectly inhibit the phosphorylation of TANK-binding kinase 1 (TBK1) by interacting with stimulator of interferon genes (STING) and glycogen synthase kinase-3β (GSK3β). This, in turn, suppresses the activation and nuclear translocation of downstream transcription factors, including interferon regulatory factor 3 (IRF3) and NF-κB. As a result, the expression of the pro-inflammatory cytokine IL-6 and the anti-inflammatory cytokine interleukin-10 (IL-10) is modulated. These regulatory effects effectively ameliorate lung injury induced by SARS-CoV-2, lipopolysaccharide (LPS), and H1N1 virus in mouse models, highlighting the therapeutic potential of inosine in inflammatory lung diseases.

**Hematological diseases**

Sickle cell anemia is an inherited blood disorder characterized by anemia resulting from an abnormal reduction in red blood cell count(Elendu et al.,2023). Notably, Kaddam et al.(Kaddam et al.,2015) demonstrated that increasing serum butyrate levels through the administration of gum arabic (GA) effectively alleviated symptoms of sickle cell anemia, highlighting a promising therapeutic avenue for the management of this disease.

Hematologic neoplasms represent a distinct class of malignancies. Notably, studies have shown that plasma arginine levels in patients with acute myeloid leukemia (AML) (~20 μM) are significantly lower than those in healthy controls (~130 μM)(Zou et al.,2024). This reduction in arginine levels contributes to tumor progression through two major mechanisms: first, by impairing immune function, as low arginine availability drives CD4⁺ T cells toward a Treg-like immunosuppressive phenotype, thereby dampening anti-tumor immune responses; and second, by promoting the upregulation of arginine metabolic pathways in tumor cells, facilitating tumor growth and immune evasion. Importantly, restoring arginine levels has been shown to significantly enhance the antitumor activity of CD8⁺ T cells in mouse models(Geiger et al.,2016). These findings suggest that moderate arginine supplementation could serve as a promising therapeutic strategy for hematologic malignancies.

**Cardiovascular diseases**

Cardiovascular disease remains the leading cause of death worldwide, accounting for up to 19.8 million deaths globally in 2022(Mensah et al.,2023). Pantothenic Acid has been shown to attenuate weight gain and significantly improve glucose tolerance and lipid metabolism disorders in obese mice fed a high-fat diet, thereby alleviating cardiovascular disease(Zhao et al.,2024). Additionally, a 4.7-year follow-up study found that higher plasma tryptophan concentrations were associated with a reduced risk of composite cardiovascular disease, suggesting that moderate dietary intake of tryptophan may help prevent cardiovascular conditions(Yu et al.,2017). Nicotinamide has also drawn increasing attention for its protective effects on the cardiovascular system. In Zucker fatty/spontaneously hypertensive heart failure F1 hybrid (ZSF1) obese rat models, it increased energy reserves in cardiac and skeletal muscles, promoted the deacetylation of troponin and Sarcoplasmic/Endoplasmic Reticulum Calcium ATPase 2a (SERCA2a), and improved both passive stiffness and active relaxation of cardiomyocytes, ultimately leading to improvements in diastolic dysfunction(Abdellatif et al.,2021). Furthermore, our metabolite–gene association network analysis revealed that the aforementioned compounds co-targeted the genes *RPS27A* and *UBA5*, suggesting that these genes may be implicated in the pathogenesis of cardiovascular disease (Figure 5C). Among them, *RPS27A* was found to be significantly upregulated at the m6A modification level, mRNA expression level, and protein level in samples from patients with idiopathic pulmonary hypertension, indicating its involvement in disease progression(Huang et al.,2023).

Hypertension is a major risk factor for cardiovascular disease. In 2023, more than 1.3 billion people worldwide are expected to be living with hypertension, with the number of affected individuals continuing to rise each year. Zhang et al. reported that Lactulose supplementation significantly reduced blood pressure in mice fed a high-salt diet. This effect is primarily attributed to the multiple mechanisms of Lactulose: on one hand, it regulates the gut microbiota, promoting the proliferation of beneficial bacteria such as bifidobacteria, which in turn enhances tryptophan metabolism and produces indole compounds with blood pressure-lowering effects; on the other hand, Lactulose reduces levels of inflammatory factors such as interleukin-17a (IL-17a) and interleukin-22 (IL-22), effectively mitigating the inflammatory response and alleviating hypertension symptoms(Zhang et al.,2019).

**Immune system diseases**

Autoimmune diseases are chronic conditions characterized by the immune system mounting an aberrant response against self-antigens. In multiple sclerosis, reduced plasma levels of phenylalanine disrupt the metabolism of tryptophan and tyrosine, leading to abnormalities in the tricarboxylic acid cycle. These metabolic disturbances impair serotonin synthesis and may contribute to increased disease susceptibility(Yu et al.,2022). Additionally, Mirzaaghasi et al. demonstrated that the combination of nicotinic acid and prednisolone exerted a synergistic effect in an experimental model of rheumatoid arthritis, resulting in improved disease outcomes(Mirzaaghasi et al.,2023).

Graves’ disease is a common autoimmune thyroid disorder, with a higher prevalence in women than in men, and is most frequently diagnosed in individuals between the ages of 20 and 60(Antonelli et al.,2020; Subekti et al.,2018). In aged female golden gophers, melatonin was shown to regulate thyroid hormone levels by reducing total triiodothyronine concentrations. Additionally, melatonin exhibits antioxidant properties by enhancing the activities of SOD, catalase, and glutathione peroxidase (GPx), while decreasing lipid peroxidation. These effects collectively help mitigate oxidative stress–induced damage caused by hyperthyroidism(Rao et al.,2016).

AIDS is a highly malignant infectious disease caused by infection with the human immunodeficiency virus(Angin et al.,2014). Due to its high mortality rate and rapid transmission, it poses a significant challenge to global public health(Pang et al.,2021). Harms et al. demonstrated that spermidine selectively inhibits X4-tropic HIV-1 infection without inducing cytotoxicity in a fetal bovine serum–free environment. Further mechanistic studies revealed that spermidine binds to the C-X-C Chemokine Receptor Type 4 (CXCR4) receptor, thereby effectively blocking X4-tropic HIV-1 entry into target cells(Harms et al.,2023).

**Endocrine diseases**

Diabetes mellitus is a prevalent chronic metabolic disorder that can be classified into type 1 diabetes (insulin-dependent), type 2 diabetes (non-insulin-dependent), gestational diabetes, and other specific types based on underlying causes. According to the Global Burden of Disease Study 2021, the global number of individuals living with diabetes has reached 529 million and is projected to exceed 1.3 billion by 2050, indicating a rapidly growing public health concern(2023). The antidiabetic potential of indole compounds has been well documented. For instance, the simple indole derivative hypaphorine has been shown to enhance insulin sensitivity, while other indole-based compounds exert antidiabetic effects through mechanisms such as inhibition of α-glucosidase and regulation of cell proliferation(Zhu et al.,2021). In support of our findings, association studies related to type 2 diabetes have also reported a role for TCA. Cheng et al. demonstrated that TCA stimulates the secretion of glucagon-like peptide-1 (GLP-1) from intestinal L-cells, thereby improving glucose metabolism by promoting insulin secretion in a glucose-dependent manner. These findings were further validated in a rat model(Cheng et al.,2018). In gestational diabetes mellitus (GDM), our association analyses revealed that daidzein, genistein, and glycitein were significantly correlated with symptom improvement, consistent with findings reported in previous studies. A prospective cohort study demonstrated that higher isoflavone intake was associated with a reduced risk of GDM, suggesting that moderate consumption of these compounds may alleviate symptoms and lower disease incidence(Dong et al.,2021). We further constructed a gene-compound association network for GDM and found that all three compounds target *MAPK1*, *MAPK3*, and *CDK4* (Figure 5D). These genes have been reported to play crucial roles in the onset and progression of GDM. Specifically, in umbilical vein endothelial cells from GDM patients, the activity of the p44/42 Mitogen-Activated Protein Kinase (MAPK) signaling pathway—including *MAPK1* and *MAPK3*—is significantly upregulated, potentially impairing placental angiogenesis and function, and thereby affecting fetal nutrient supply(Subiabre et al.,2017). Additionally, *CDK4* is downregulated in the GDM setting, leading to impaired pancreatic β-cell proliferation and reduced insulin secretion, which may hinder fetal development and increase the risk of diabetes in offspring(Nazari et al.,2017).

Thyroid diseases, a common endocrine disorder, show increasing prevalence with age and are particularly prominent among the elderly(Faggiano et al.,2011). In a rat model of hyperthyroidism, leucine supplementation improved exercise endurance and reduced circulating levels of creatine kinase-MB (CK-MB), a biomarker of muscle injury(Fidale et al.,2018). These effects suggest that leucine may help mitigate functional impairments caused by thyroid dysfunction.

Metabolic syndrome is a complex condition characterized by disturbed metabolism of proteins, fats, and other substances. In a study involving high-fat diet-induced obese mice, intraperitoneal injection of spermine reduced body weight and blood glucose levels while enhancing glucose utilization, a series of changes that contributed to alleviating symptoms associated with metabolic syndrome(Sadasivan et al.,2014). Baxheinrich et al. demonstrated that an energy-restricted diet rich in canola oil, which is high in alpha-linolenic acid (ALA), resulted in a more significant risk reduction for metabolic syndrome than an energy-restricted diet rich in olive oil(Baxheinrich et al.,2012).

**Oral Diseases**

Gingivitis is the most common gingival disease. Previous studies suggest that D-Arabinose acts as a quorum-sensing inhibitor and plays vital role in the control of periodontal disease. It inhibits biofilm formation and the activity of autoinducer 2 in periodontal pathogens. In the THP-1 monocyte assay, outer membrane vesicles of *Porphyromonas gingivalis* treated with D-Arabinose significantly reduced the production of pro-inflammatory factors such as Tumor Necrosis Factor-alpha (TNF-α) and Interleukin-1 beta (IL-1β). Additionally, D-Arabinose reduced the activation of Toll-like Receptor 2 (TLR2), effectively decreasing the pro-inflammatory activity of the *Porphyromonas gingivalis* outer membrane vesicles and thereby inhibiting the progression of periodontal disease(An et al.,2023).

**Neurological diseases**

Alzheimer’s disease (AD) is a progressive neurodegenerative disorder of the central nervous system. Pathological mechanisms contributing to cognitive decline in AD include β-amyloid (Aβ) deposition, hyperphosphorylation of Tau protein leading to the formation of intracellular neurofibrillary tangles, and impaired adult hippocampal neurogenesis(Bai et al.,2022; Ying et al.,2021). In an animal model, intervention with 3-hydroxybutyric acid was shown to improve cognitive function, reduce Aβ accumulation and microglial hyperactivation in the brain, and enhance mitochondrial respiration in hippocampal neurons. These effects collectively protected neurons from Aβ-induced toxicity, thereby contributing to neuronal restoration(Mierziak et al.,2021).

Migraine is a prevalent chronic neurovascular disorder affecting over one billion people worldwide, with an annual prevalence rate of approximately 15%, making it a significant global public health issue(Ashina et al.,2021). A case report documented a patient whose migraine symptoms responded markedly to prophylactic treatment with extended-release niacin(Velling et al.,2003). It has been hypothesized that niacin may exert therapeutic effects through vasodilation and enhancement of mitochondrial energy metabolism. However, its precise mechanism of action has not yet been fully validated in controlled clinical trials, and further experimental studies are required to determine its clinical relevance(Prousky et al.,2005).

Autism is a common neurodevelopmental disorder, and its etiology remains inconclusive. Castejon et al. conducted a randomized, double-blind, controlled study involving 46 children with autism aged 3–5 years, of whom 40 completed a 90-day treatment course. The results showed that children receiving a cysteine-enriched casein-rich whey protein (CRWP) formulation exhibited significantly elevated glutathione levels (p = 0.04) and notable improvements in multiple domains of the Vineland Adaptive Behavior Scales, Second Edition (VABS-II), including a significant increase in the composite score (p = 0.03), suggesting its potential efficacy in improving autism-related behaviors(Castejon et al.,2021).

Bipolar disorder is a common psychiatric condition affecting approximately 1–2% of the global population, characterized by recurrent episodes of mania and depression(Burdick et al.,2023; Smith et al.,2012). Notably, Gurevich et al. retrospectively analyzed clinical cases and found that both leucine and l-isoleucine are components of a branched-chain amino acid (BCAA) formulation used to alleviate bipolar disorder symptoms. When administered alongside other supplements, BCAA effectively protected most patients from hypomanic episodes(Gurevich et al.,2016). Additionally, experimental studies have demonstrated that phenylalanine can elevate mood in the majority of patients with depression, and its metabolite, phenylacetic acid (PAA), has been shown to correlate with depressive states(Sabelli et al.,1986). Further analysis of the metabolite–gene association network revealed that these three metabolites collectively target the *PSMD14* gene (Supplementary Figure 1). Interestingly, recent studies have implicated *PSMD14* in the pathogenesis of bipolar disorder and suggested its potential as a therapeutic target(O'Connell et al.,2025).

**Other disease types**

Cystic fibrosis is an autosomal recessive disorder that typically presents clinically with gastrointestinal and respiratory abnormalities(Liessi et al.,2020). Kim et al. found that Stearic Acid levels in patients with idiopathic pulmonary fibrosis were significantly lower than those in healthy controls, as revealed by metabolomics analysis. Furthermore, both in vitro and in vivo experiments demonstrated that Stearic Acid could inhibit the transforming growth factor-β1 (TGF-β1)-induced activation of the Smad2/3 pathway and the generation of reactive oxygen species (ROS), thereby decreasing the expression of fibrosis-associated proteins and exhibiting its antifibrotic effects(Kim et al.,2021). Silva et al. found that, in their study of lung infections in patients with cystic fibrosis, Stearic Acid levels were significantly lower compared to those in healthy controls. Pseudomonas aeruginosa infections are difficult to eradicate in the cystic fibrosis airway environment, and Aspartic Acid has shown potential to improve the associated therapy. In vitro experiments demonstrated that Aspartic Acid enhances the antimicrobial activity of ciprofloxacin against *Pseudomonas aeruginosa* when combined with the antibiotic(Silva et al.,2020).

**Supplementary Figures**

**
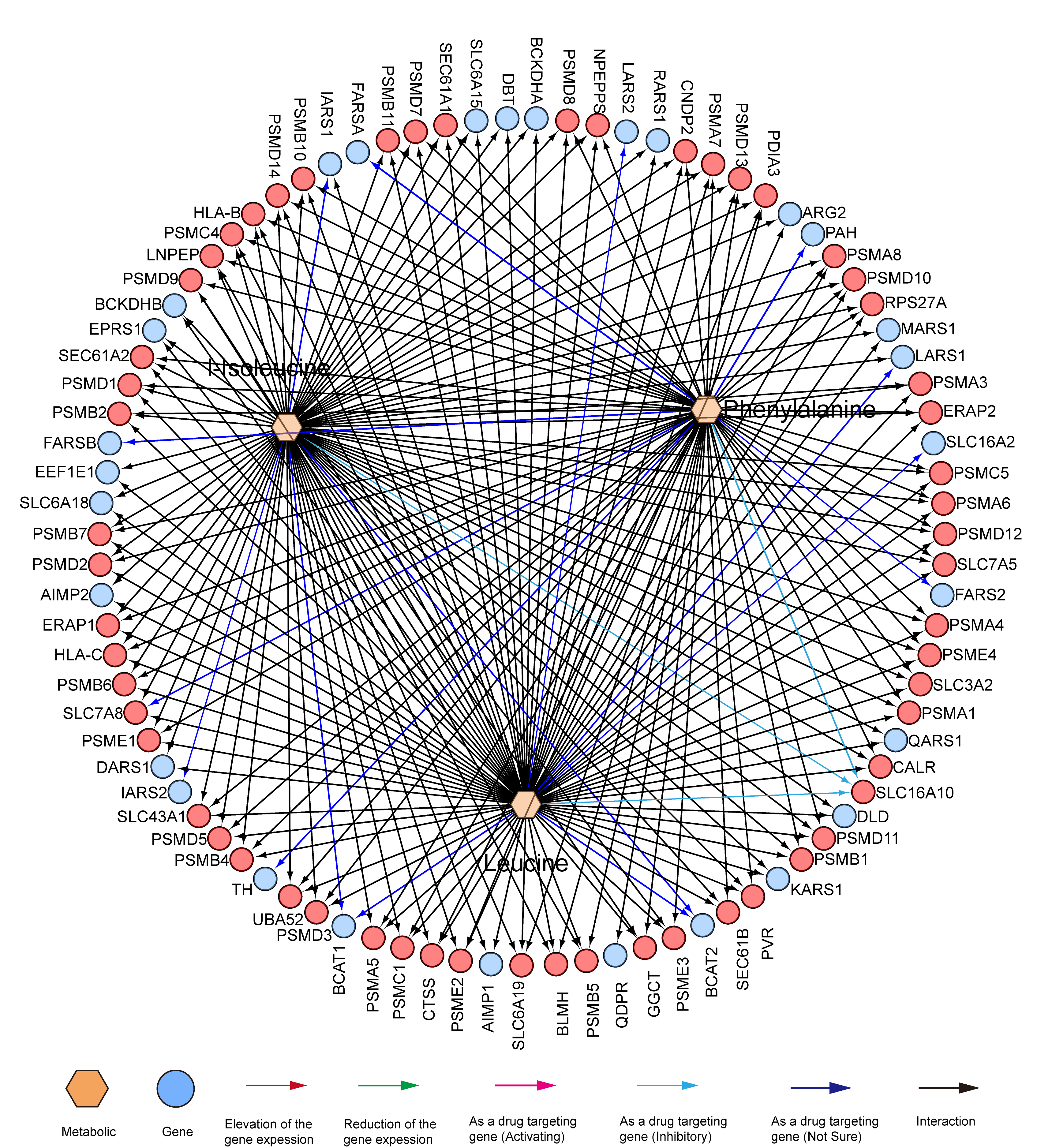
**

**Supplementary Fig. 1** | **Metabolite gene association network in bipolar disorder.** The red nodes represent genes that are jointly targeted by three metabolites.


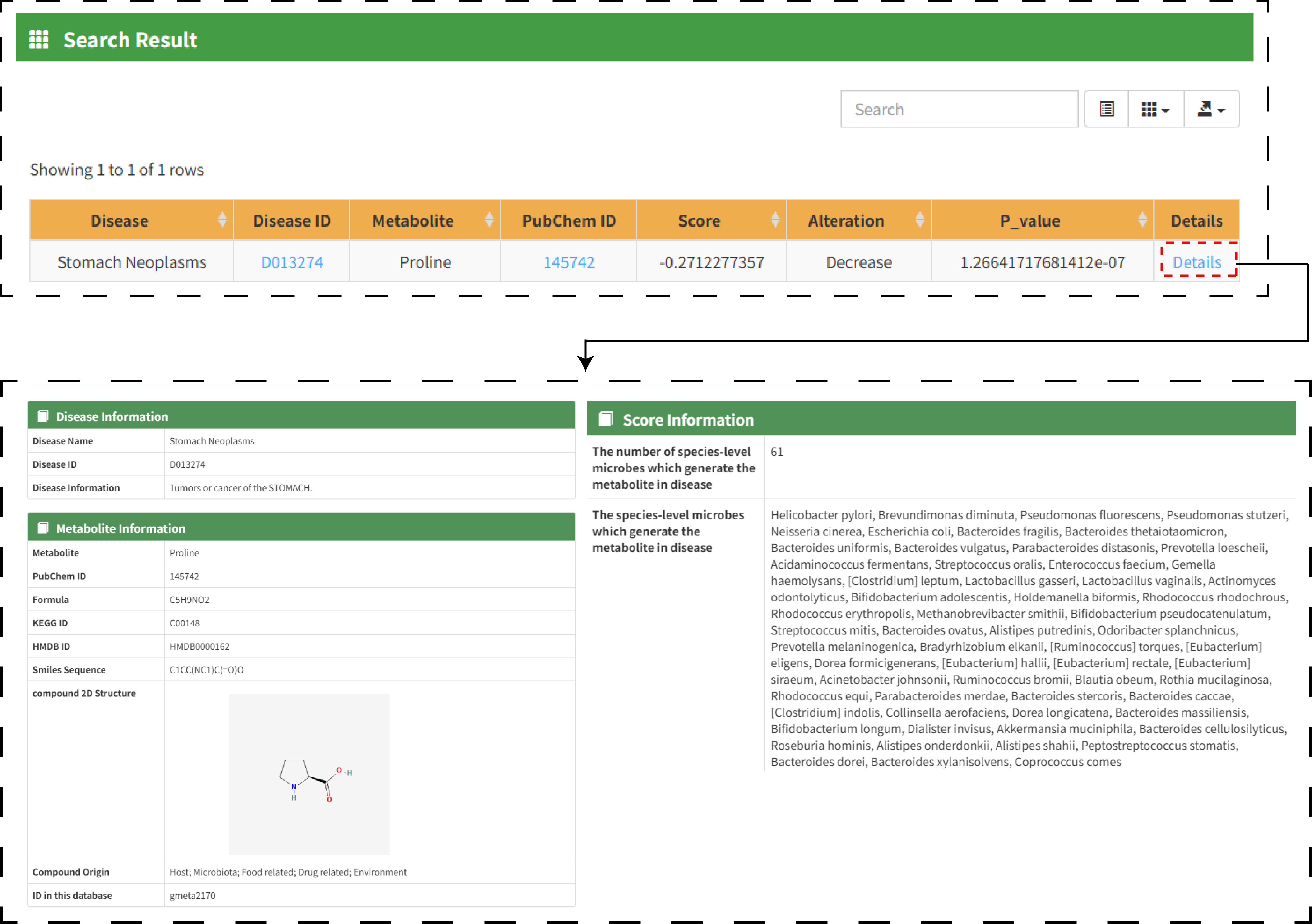


**Supplementary Fig. 2** | **Search results for Stomach Neoplasms disease and Proline metabolite.**

**References:**

2023). Global, regional, and national burden of diabetes from 1990 to 2021, with projections of prevalence to 2050: a systematic analysis for the global burden of disease study 2021. *Lancet*. 402(10397), 203-234. doi: 10.1016/S0140-6736(23)01301-6

Abdellatif, M., Trummer-Herbst, V., Koser, F., Durand, S., Adao, R., and Vasques-Novoa, F., et al. (2021). Nicotinamide for the treatment of heart failure with preserved ejection fraction. *Sci. Transl. Med.* 13(580). doi: 10.1126/scitranslmed.abd7064

An, S. J., Ha, K. W., Jun, H. K., Kim, H. Y., Choi, B. K. (2023). Reduced proinflammatory activity of outer membrane vesicles of tannerella forsythia treated with quorum sensing inhibitors. *Mol. Oral Microbiol.* 38(1), 71-81. doi: 10.1111/omi.12380

Andrade, M. E. R., de Barros, P. A. V., Menta, P. L. D. R., Costa, G. M. F., Miranda, S. E. M., and Leocadio, P. C. L., et al. (2019). Arginine supplementation reduces colonic injury, inflammation and oxidative stress of dss-induced colitis in mice. *J. Funct. Foods*. 52, 360-369. doi: 10.1016/j.jff.2018.11.019

Angin, M., Sharma, S., King, M., Murooka, T. T., Ghebremichael, M., and Mempel, T. R., et al. (2014). Hiv-1 infection impairs regulatory t-cell suppressive capacity on a per-cell basis. *J. Infect. Dis.* 210(6), 899-903. doi: 10.1093/infdis/jiu188

Antonelli, A., Ferrari, S. M., Ragusa, F., Elia, G., Paparo, S. R., and Ruffilli, I., et al. (2020). Graves' disease: epidemiology, genetic and environmental risk factors and viruses. *Best Pract. Res. Clin. Endoc. Metab.* 34(1), 101387. doi: 10.1016/j.beem.2020.101387

Arteh, J., Narra, S., Nair, S. (2010). Prevalence of vitamin d deficiency in chronic liver disease. *Dig. Dis. Sci.* 55(9), 2624-2628. doi: 10.1007/s10620-009-1069-9

Ashina, M., Katsarava, Z., Do, T. P., Buse, D. C., Pozo-Rosich, P., and Ozge, A., et al. (2021). Migraine: epidemiology and systems of care. *Lancet*. 397(10283), 1485-1495. doi: 10.1016/S0140-6736(20)32160-7

Bai, R., Guo, J., Ye, X. Y., Xie, Y., Xie, T. (2022). Oxidative stress: the core pathogenesis and mechanism of alzheimer's disease. *Ageing Res. Rev.* 77, 101619. doi: 10.1016/j.arr.2022.101619

Baxheinrich, A., Stratmann, B., Lee-Barkey, Y. H., Tschoepe, D., Wahrburg, U. (2012). Effects of a rapeseed oil-enriched hypoenergetic diet with a high content of alpha-linolenic acid on body weight and cardiovascular risk profile in patients with the metabolic syndrome. *Br. J. Nutr.* 108(4), 682-691. doi: 10.1017/S0007114512002875

Bray, F., Laversanne, M., Sung, H., Ferlay, J., Siegel, R. L., and Soerjomataram, I., et al. (2024). Global cancer statistics 2022: globocan estimates of incidence and mortality worldwide for 36 cancers in 185 countries. *Ca. Cancer. J. Clin.* 74(3), 229-263. doi: 10.3322/caac.21834

Burdick, K. E., Millett, C. E., Yocum, A. K., Altimus, C. M., Andreassen, O. A., and Aubin, V., et al. (2023). Predictors of functional impairment in bipolar disorder: results from 13 cohorts from seven countries by the global bipolar cohort collaborative. *Focus (Am Psychiatr Publ)*. 21(4), 444-452. doi: 10.1176/appi.focus.23021021

Castejon, A. M., Spaw, J. A., Rozenfeld, I., Sheinberg, N., Kabot, S., and Shaw, A., et al. (2021). Improving antioxidant capacity in children with autism: a randomized, double-blind controlled study with cysteine-rich whey protein. *Front. Psychiatry*. 12, 669089. doi: 10.3389/fpsyt.2021.669089

Chapelle, N., Ben, G. I., Barkun, A., Bardou, M. (2021). The pharmacotherapeutic management of gastroesophageal reflux disease (gerd). *Expert Opin. Pharmacother.* 22(2), 219-227. doi: 10.1080/14656566.2020.1817385

Cheng, Z., Liu, G., Zhang, X., Bi, D., Hu, S. (2018). Improvement of glucose metabolism following long-term taurocholic acid gavage in a diabetic rat model. *Med. Sci. Monitor*. 24, 7206-7212. doi: 10.12659/MSM.912429

Dior, M., Delagreverie, H., Duboc, H., Jouet, P., Coffin, B., and Brot, L., et al. (2016). Interplay between bile acid metabolism and microbiota in irritable bowel syndrome. *Neurogastroenterol. Motil.* 28(9), 1330-1340. doi: 10.1111/nmo.12829

Dong, J. Y., Kimura, T., Ikehara, S., Cui, M., Kawanishi, Y., and Kimura, T., et al. (2021). Soy consumption and incidence of gestational diabetes mellitus: the japan environment and children's study. *Eur. J. Nutr.* 60(2), 897-904. doi: 10.1007/s00394-020-02294-1

Elendu, C., Amaechi, D. C., Alakwe-Ojimba, C. E., Elendu, T. C., Elendu, R. C., and Ayabazu, C. P., et al. (2023). Understanding sickle cell disease: causes, symptoms, and treatment options. *Medicine. (Baltimore).* 102(38), e35237. doi: 10.1097/MD.0000000000035237

Faggiano, A., Del, P. M., Marciello, F., Marotta, V., Ramundo, V., and Colao, A. (2011). Thyroid diseases in elderly. *Minerva Endocrinol.* 36(3), 211-231

Fidale, T. M., Antunes, H., Roever, L., Goncalves, A., Puga, G. M., and Silva, R., et al. (2018). Leucine supplementation improves effort tolerance of rats with hyperthyroidism. *Front. Physiol.* 9, 1632. doi: 10.3389/fphys.2018.01632

Gan, C., Yuan, Y., Shen, H., Gao, J., Kong, X., and Che, Z., et al. (2025). Liver diseases: epidemiology, causes, trends and predictions. *Signal Transduct. Target. Ther.* 10(1), 33. doi: 10.1038/s41392-024-02072-z

Geiger, R., Rieckmann, J. C., Wolf, T., Basso, C., Feng, Y., and Fuhrer, T., et al. (2016). L-arginine modulates t cell metabolism and enhances survival and anti-tumor activity. *Cell*. 167(3), 829-842. doi: 10.1016/j.cell.2016.09.031

Gu, Y., Chen, T., Fu, S., Sun, X., Wang, L., and Wang, J., et al. (2015). Perioperative dynamics and significance of amino acid profiles in patients with cancer. *J. Transl. Med.* 13, 35. doi: 10.1186/s12967-015-0408-1

Gurevich, M. I., Robinson, C. L. (2016). An individualized approach to treatment-resistant bipolar disorder: a case series. *Explore-J Sci. Heal.* 12(4), 237-245. doi: 10.1016/j.explore.2016.04.001

Halestrap, A. P. (2013). The slc16 gene family - structure, role and regulation in health and disease. *Mol. Aspects. Med.* 34(2-3), 337-349. doi: 10.1016/j.mam.2012.05.003

Haraguchi, T., Kayashima, T., Okazaki, Y., Inoue, J., Mineo, S., and Matsubara, K., et al. (2014). Cecal succinate elevated by some dietary polyphenols may inhibit colon cancer cell proliferation and angiogenesis. *J. Agric. Food. Chem.* 62(24), 5589-5594. doi: 10.1021/jf501142k

Harms, M., Smith, N., Han, M., Gross, R., von Maltitz, P., and Sturzel, C., et al. (2023). Spermine and spermidine bind cxcr4 and inhibit cxcr4- but not ccr5-tropic hiv-1 infection. *Sci. Adv.* 9(27), eadf8251. doi: 10.1126/sciadv.adf8251

Huang, G. R., Wei, S. J., Huang, Y. Q., Xing, W., Wang, L. Y., and Liang, L. L. (2018). Mechanism of combined use of vitamin d and puerarin in anti-hepatic fibrosis by regulating the wnt/beta-catenin signalling pathway. *World J. Gastroenterol.* 24(36), 4178-4185. doi: 10.3748/wjg.v24.i36.4178

Huang, T., Zeng, Y., Yang, Y., Fan, H., Deng, Y., and Chen, W., et al. (2023). Comprehensive analysis of m(6)a methylomes in idiopathic pulmonary arterial hypertension. *Epigenetics*. 18(1), 2242225. doi: 10.1080/15592294.2023.2242225

Kaddam, L., FdleAlmula, I., Eisawi, O. A., Abdelrazig, H. A., Elnimeiri, M., and Lang, F., et al. (2015). Gum arabic as fetal hemoglobin inducing agent in sickle cell anemia; In vivo study. *Bmc Hematol*. 15, 19. doi: 10.1186/s12878-015-0040-6

Kim, H. S., Yoo, H. J., Lee, K. M., Song, H. E., Kim, S. J., and Lee, J. O., et al. (2021). Stearic acid attenuates profibrotic signalling in idiopathic pulmonary fibrosis. *Respirology*. 26(3), 255-263. doi: 10.1111/resp.13949

Kozmus, C. E., Moura, E., Serrao, M. P., Real, H., Guimaraes, J. T., and Guedes-de-Pinho, P., et al. (2011). Influence of dietary supplementation with dextrin or oligofructose on the hepatic redox balance in rats. *Mol. Nutr. Food Res.* 55(11), 1735-1739. doi: 10.1002/mnfr.201100287

Li, J., Yan, Y., Fu, Y., Chen, Z., Yang, Y., and Li, Y., et al. (2024). Ace2 mediates tryptophan alleviation on diarrhea by repairing intestine barrier involved mtor pathway. *Cell. Mol. Biol. Lett.* 29(1), 90. doi: 10.1186/s11658-024-00603-8

Li, S., Zhou, T., Li, C., Dai, Z., Che, D., and Yao, Y., et al. (2014). High metastaticgastric and breast cancer cells consume oleic acid in an ampk dependent manner. *Plos One*. 9(5), e97330. doi: 10.1371/journal.pone.0097330

Liessi, N., Pedemonte, N., Armirotti, A., Braccia, C. (2020). Proteomics and metabolomics for cystic fibrosis research. *Int. J. Mol. Sci.* 21(15). doi: 10.3390/ijms21155439

Lin, C. C., Hsieh, N. K., Liou, H. L., Chen, H. I. (2012). Niacinamide mitigated the acute lung injury induced by phorbol myristate acetate in isolated rat's lungs. *J. Biomed. Sci.* 19(1), 27. doi: 10.1186/1423-0127-19-27

Mariotta, L., Ramadan, T., Singer, D., Guetg, A., Herzog, B., and Stoeger, C., et al. (2012). T-type amino acid transporter tat1 (slc16a10) is essential for extracellular aromatic amino acid homeostasis control. *J. Physiol.* 590(24), 6413-6424. doi: 10.1113/jphysiol.2012.239574

Mensah, G. A., Fuster, V., Roth, G. A. (2023). A heart-healthy and stroke-free world: using data to inform global action. *J. Am. Coll. Cardiol.* 82(25), 2343-2349. doi: 10.1016/j.jacc.2023.11.003

Mierziak, J., Burgberger, M., Wojtasik, W. (2021). 3-hydroxybutyrate as a metabolite and a signal molecule regulating processes of living organisms. *Biomolecules*. 11(3). doi: 10.3390/biom11030402

Mirzaaghasi, S., Froushani, S. (2023). Immunomodulatory effects of combined nicotinic acid and prednisolone in adjuvant-induced arthritis. *Antiinflamm Antiallergy Agents Med Chem*. 22(2), 104-112. doi: 10.2174/0118715230264101230925060355

Nagahama, K., Nishio, H., Yamato, M., Takeuchi, K. (2012). Orally administered l-arginine and glycine are highly effective against acid reflux esophagitis in rats. *Med. Sci. Monitor*. 18(1), BR9-BR15. doi: 10.12659/msm.882190

Nanthakumaran, S., Brown, I., Heys, S. D., Schofield, A. C. (2009). Inhibition of gastric cancer cell growth by arginine: molecular mechanisms of action. *Clin. Nutr.* 28(1), 65-70. doi: 10.1016/j.clnu.2008.10.007

Nazari, Z., Nabiuni, M., Saeidi, M., Golalipour, M. J. (2017). Gestational diabetes leads to down-regulation of cdk4-prb-e2f1 pathway genes in pancreatic islets of rat offspring. *Iran. J. Basic Med. Sci.* 20(2), 150-154. doi: 10.22038/ijbms.2017.8240

O'Connell, K. S., Koromina, M., van der Veen, T., Boltz, T., David, F. S., and Yang, J., et al. (2025). Genomics yields biological and phenotypic insights into bipolar disorder. *Nature*. 639(8056), 968-975. doi: 10.1038/s41586-024-08468-9

Otto, J., Verwaayen, A., Penners, C., Hundertmark, J., Lin, C., and Kallen, C., et al. (2023). Expression of cyclin e1 in hepatic stellate cells is critical for the induction and progression of liver fibrosis and hepatocellular carcinoma in mice. *Cell Death Dis.* 14(8), 549. doi: 10.1038/s41419-023-06077-4

Pang, X., Wei, H., Huang, J., He, Q., Tang, K., and Fang, N., et al. (2021). Patterns and risk of hiv-1 transmission network among men who have sex with men in guangxi, china. *Sci. Rep.* 11(1), 513. doi: 10.1038/s41598-020-79951-2

Prousky, J., Seely, D. (2005). The treatment of migraines and tension-type headaches with intravenous and oral niacin (nicotinic acid): systematic review of the literature. *Nutr. J.* 4, 3. doi: 10.1186/1475-2891-4-3

Rao, G., Verma, R., Mukherjee, A., Haldar, C., Agrawal, N. K. (2016). Melatonin alleviates hyperthyroidism induced oxidative stress and neuronal cell death in hippocampus of aged female golden hamster, mesocricetus auratus. *Exp. Gerontol.* 82, 125-130. doi: 10.1016/j.exger.2016.06.014

Ribeiro, J. H. C., Lifshitz, F. (1991). Alanine-based oral rehydration therapy for infants with acute diarrhea. *J. Pediatr.* 118(4 Pt 2), S86-S90. doi: 10.1016/s0022-3476(05)81432-5

Sabelli, H. C., Fawcett, J., Gusovsky, F., Javaid, J. I., Wynn, P., and Edwards, J., et al. (1986). Clinical studies on the phenylethylamine hypothesis of affective disorder: urine and blood phenylacetic acid and phenylalanine dietary supplements. *J. Clin. Psychiatry.* 47(2), 66-70

Sadasivan, S. K., Vasamsetti, B., Singh, J., Marikunte, V. V., Oommen, A. M., and Jagannath, M. R., et al. (2014). Exogenous administration of spermine improves glucose utilization and decreases bodyweight in mice. *Eur. J. Pharmacol.* 729, 94-99. doi: 10.1016/j.ejphar.2014.01.073

Selamnia, M., Robert, V., Mayeur, C., Duee, P. H., Blachier, F. (1998). Effects of l-valine on growth and polyamine metabolism in human colon carcinoma cells. *Biochim Biophys Acta*. 1379(1), 151-160. doi: 10.1016/s0304-4165(97)00095-0

Silva, E., Monteiro, R., Grainha, T., Alves, D., Pereira, M. O., and Sousa, A. M. (2020). Fostering innovation in the treatment of chronic polymicrobial cystic fibrosis-associated infections exploring aspartic acid and succinic acid as ciprofloxacin adjuvants. *Front. Cell. Infect. Microbiol.* 10, 441. doi: 10.3389/fcimb.2020.00441

Smith, D. J., Whitham, E. A., Ghaemi, S. N. (2012). Bipolar disorder. *Handb Clin Neurol*. 106, 251-263. doi: 10.1016/B978-0-444-52002-9.00015-2

Subekti, I., Pramono, L. A. (2018). Current diagnosis and management of graves' disease. *Acta Med. Indones.* 50(2), 177-182

Subiabre, M., Silva, L., Villalobos-Labra, R., Toledo, F., Paublo, M., and Lopez, M. A., et al. (2017). Maternal insulin therapy does not restore foetoplacental endothelial dysfunction in gestational diabetes mellitus. *Biochim. Biophys. Acta-Mol. Basis Dis.* 1863(11), 2987-2998. doi: 10.1016/j.bbadis.2017.07.022

Sun, S., Xu, M., Zhuang, P., Chen, G., Dong, K., and Dong, R., et al. (2021). Effect and mechanism of vitamin d activation disorder on liver fibrosis in biliary atresia. *Sci. Rep.* 11(1), 19883. doi: 10.1038/s41598-021-99158-3

Thapaliya, S., Wree, A., Povero, D., Inzaugarat, M. E., Berk, M., and Dixon, L., et al. (2014). Caspase 3 inactivation protects against hepatic cell death and ameliorates fibrogenesis in a diet-induced nash model. *Dig. Dis. Sci.* 59(6), 1197-1206. doi: 10.1007/s10620-014-3167-6

Turjman, N., Gotterer, G. S., Hendrix, T. R. (1978). Prevention and reversal of cholera enterotoxin effects in rabbit jejunum by nicotinic acid. *J. Clin. Invest.* 61(5), 1155-1160. doi: 10.1172/JCI109030

Ubaid, M., Salauddin, Shadani, M. A., Kawish, S. M., Albratty, M., and Makeen, H. A., et al. (2023). Daidzein from dietary supplement to a drug candidate: an evaluation of potential. *Acs Omega*. 8(36), 32271-32293. doi: 10.1021/acsomega.3c03741

Velling, D. A., Dodick, D. W., Muir, J. J. (2003). Sustained-release niacin for prevention of migraine headache. *Mayo. Clin. Proc.* 78(6), 770-771. doi: 10.4065/78.6.770

Vijayvargiya, P., Busciglio, I., Burton, D., Donato, L., Lueke, A., and Camilleri, M. (2018). Bile acid deficiency in a subgroup of patients with irritable bowel syndrome with constipation based on biomarkers in serum and fecal samples. *Clin. Gastroenterol. Hepatol.* 16(4), 522-527. doi: 10.1016/j.cgh.2017.06.039

Wang, N., Li, E., Deng, H., Yue, L., Zhou, L., and Su, R., et al. (2023). Inosine: a broad-spectrum anti-inflammatory against SARS-cov-2 infection-induced acute lung injury via suppressing tbk1 phosphorylation. *J. Pharm. Anal.* 13(1), 11-23. doi: 10.1016/j.jpha.2022.10.002

Wang, Y., Jia, Z., Wang, Q., Zhu, Z. (2023). Amino acids and risk of colon adenocarcinoma: a mendelian randomization study. *Bmc Cancer*. 23(1), 1041. doi: 10.1186/s12885-023-11514-w

Xu, J., Xie, S., Chi, S., Zhang, S., Cao, J., and Tan, B. (2022). Protective effects of taurocholic acid on excessive hepatic lipid accumulation via regulation of bile acid metabolism in grouper. *Food Funct.* 13(5), 3050-3062. doi: 10.1039/d1fo04085e

Ying, Y., Wang, J. Z. (2021). Illuminating neural circuits in alzheimer's disease. *Neurosci. Bull.* 37(8), 1203-1217. doi: 10.1007/s12264-021-00716-6

Yu, E., Ruiz-Canela, M., Guasch-Ferre, M., Zheng, Y., Toledo, E., and Clish, C. B., et al. (2017). Increases in plasma tryptophan are inversely associated with incident cardiovascular disease in the prevencion con dieta mediterranea (predimed) study. *J. Nutr.* 147(3), 314-322. doi: 10.3945/jn.116.241711

Yu, X. H., Cao, R. R., Yang, Y. Q., Lei, S. F. (2022). Identification of causal metabolites related to multiple autoimmune diseases. *Hum. Mol. Genet.* 31(4), 604-613. doi: 10.1093/hmg/ddab273

Zhang, B., Liu, M., Yue, Z., Chen, X., Li, C., and Liu, L., et al. (2023). Combined omics analysis further unveils the specific role of butyrate in promoting growth in early-weaning animals. *Int. J. Mol. Sci.* 24(2). doi: 10.3390/ijms24021787

Zhang, Y., Tu, S., Ji, X., Wu, J., Meng, J., and Gao, J., et al. (2024). Dubosiella newyorkensis modulates immune tolerance in colitis via the l-lysine-activated ahr-ido1-kyn pathway. *Nat. Commun.* 15(1), 1333. doi: 10.1038/s41467-024-45636-x

Zhang, Z., Zhao, J., Tian, C., Chen, X., Li, H., and Wei, X., et al. (2019). Targeting the gut microbiota to investigate the mechanism of lactulose in negating the effects of a high-salt diet on hypertension. *Mol. Nutr. Food Res.* 63(11), e1800941. doi: 10.1002/mnfr.201800941

Zhao, C., Wen, Z., Gao, Y., Xiao, F., Yan, J., and Wang, X., et al. (2024). Pantothenic acid alleviates fat deposition and inflammation by suppressing the jnk/p38 mapk signaling pathway. *J. Med. Food*. 27(9), 834-843. doi: 10.1089/jmf.2023.k.0292

Zhu, Y., Zhao, J., Luo, L., Gao, Y., Bao, H., and Li, P., et al. (2021). Research progress of indole compounds with potential antidiabetic activity. *Eur. J. Med. Chem.* 223, 113665. doi: 10.1016/j.ejmech.2021.113665

Zou, Z., Cheng, Q., Zhou, J., Guo, C., Hadjinicolaou, A. V., and Salio, M., et al. (2024). Atf4-slc7a11-gsh axis mediates the acquisition of immunosuppressive properties by activated cd4(+) t cells in low arginine condition. *Cell Rep.* 43(4), 113995. doi: 10.1016/j.celrep.2024.113995
